# New pathogenic *ZNHIT3* variants causing fetal hydrops and lethality disrupt snoRNP assembly

**DOI:** 10.1093/nar/gkag1024

**Published:** 2026-11-05

**Authors:** Md Lutfur Rahman, Adeline A Bonnard, Lyse Ruaud, Fabien Guimiot, Ines Defer, Feng Wang, Virginie Marchand, Yuri Motorin, Bing Yao, Séverine Drunat, Homa Ghalei

**Affiliations:** Department of Biochemistry, Emory University School of Medicine, Atlanta, GA 30322, United States; Génétique du développement, Hôpital Robert Debré, Assistance Publique des Hôpitaux de Paris (AP-HP), 75019 Paris, France; INSERM UMR 1141, Paris-Cité University, NeuroDiderot, Paris 75019, France; Génétique du développement, Hôpital Robert Debré, Assistance Publique des Hôpitaux de Paris (AP-HP), 75019 Paris, France; INSERM UMR 1141, Paris-Cité University, NeuroDiderot, Paris 75019, France; Génétique du développement, Hôpital Robert Debré, Assistance Publique des Hôpitaux de Paris (AP-HP), 75019 Paris, France; INSERM UMR 1141, Paris-Cité University, NeuroDiderot, Paris 75019, France; Génétique du développement, Hôpital Robert Debré, Assistance Publique des Hôpitaux de Paris (AP-HP), 75019 Paris, France; INSERM UMR 1141, Paris-Cité University, NeuroDiderot, Paris 75019, France; Department of Human Genetics, Emory University School of Medicine, Atlanta, GA 30322, United States; Université de Lorraine, SMP IBSLor, Biopôle, 9 Avenue de la Forêt de Haye, 54505 Vandoeuvre-les-Nancy, France; Université de Lorraine, UMR7365 IMoPA, CNRS, Biopôle, 9 Avenue de la Forêt de Haye, 54505 Vandoeuvre-les-Nancy, France; Department of Human Genetics, Emory University School of Medicine, Atlanta, GA 30322, United States; Génétique du développement, Hôpital Robert Debré, Assistance Publique des Hôpitaux de Paris (AP-HP), 75019 Paris, France; INSERM UMR 1141, Paris-Cité University, NeuroDiderot, Paris 75019, France; Department of Biochemistry, Emory University School of Medicine, Atlanta, GA 30322, United States

## Abstract

ZNHIT3 is a conserved assembly factor required for biogenesis of box C/D small nucleolar ribonucleoprotein (snoRNP) complexes, which are key players in ribosome biogenesis. Pathogenic variants in *ZNHIT3* cause Progressive Encephalopathy with Edema, Hypsarrhythmia, and Optic Atrophy (PEHO) syndrome, a severe neurodevelopmental disorder typically recognized postnatally. Here, we describe two fetuses from a single family who developed isolated hydrops in the early second trimester of pregnancy, resulting in intrauterine demise. Whole-genome quartet sequencing identified two new *ZNHIT3* variants, c.40T > C; p.(Cys14Arg) and c.251_254del; p.(Glu84Alafs*8), inherited from healthy parents that present as compound heterozygous alleles in both fetuses. Functional studies using a *ZNHIT3* knockdown-rescue system in HEK293T cells demonstrated impaired proliferation and increased apoptosis and revealed distinct molecular consequences for the two variants. One variant produced a stable ZNHIT3 protein lacking the region required for interaction with NUFIP1 and efficient box C/D snoRNP assembly, whereas the second variant reduced ZNHIT3 protein abundance. Both variants impaired snoRNP assembly, reduced the abundance of select box C/D snoRNAs, and caused site-specific losses of ribosomal RNA (rRNA) 2′-O-methylation. These defects were accompanied by changes in ribosome biogenesis and reduced translational output. Together, our findings expand the phenotypic spectrum associated with ZNHIT3 deficiency to include severe antenatal disease and demonstrate that impaired box C/D snoRNP assembly and rRNA modification represent convergent molecular consequences of pathogenic *ZNHIT3* variants.

## Graphical Abstract

**Graphical Abstract:**
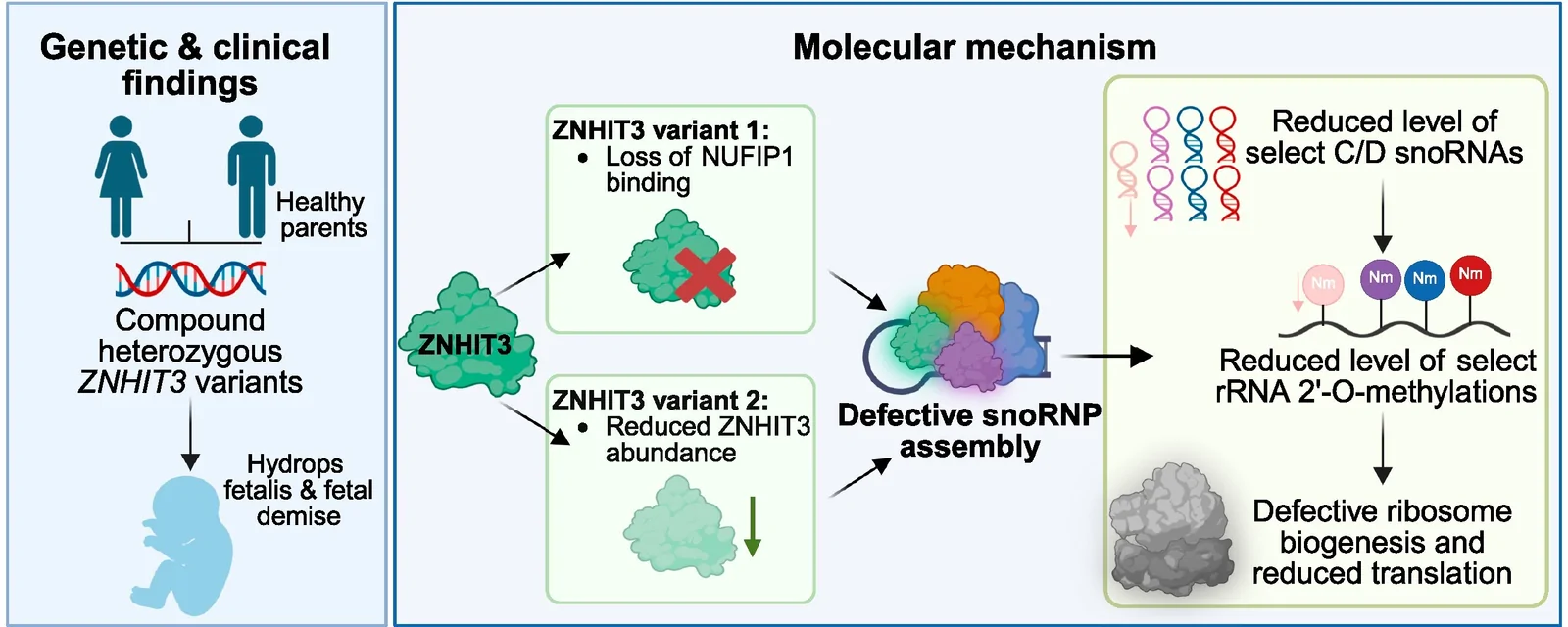

## Introduction

Regulation of protein homeostasis is crucial for cell survival and development across all domains of life. Therefore, the production of ribosomes, responsible for protein synthesis, is tightly regulated by a large network of ribosome biogenesis factors, including proteins and noncoding RNAs [1]. A critical step in ribosome biogenesis involves the synthesis, processing, folding, and chemical modification of ribosomal RNAs (rRNAs) [2–4]. Highly abundant small nucleolar RNAs (snoRNAs) are required for rRNA processing and guide the majority of rRNA chemical modifications in eukaryotes [5, 6]. Dysregulation of snoRNA levels or their function is associated with human diseases ranging from cancer to immunodeficiency, metabolic disorders, and neurological disorders [7–15].

snoRNAs are classified into two primary types based on their conserved sequence motifs. SNORDs, characterized by their conserved C/D and C’/D’ sequence motifs, primarily guide 2′-O-methylation [16], while two of them are involved in guiding rRNA acetylation [17]. The second major group, SNORAs, characterized by their conserved box H/ACA elements, primarily guide the conversion of uridines to pseudouridines [18]. Besides those involved in chemical modifications, several snoRNAs are essential for rRNA processing and cleavage, some are involved in other cellular activities, and a subset has no known targets or functions and is therefore considered orphans [19].

snoRNAs associate with a set of evolutionarily conserved core proteins to form small nucleolar ribonucleoprotein (snoRNP) complexes that guide and catalyze site-specific modifications of rRNAs. Box C/D snoRNPs comprise four essential core proteins: SNU13, NOP56, NOP58, and the 2′-O-methyltransferase fibrillarin [20]. Consistent with their essential roles, disease-causing variants in core snoRNP components have been reported. A recently reported *NOP58* disease variant was associated with reduced box C/D snoRNA levels and defects in pre-rRNA maturation, implicating box C/D snoRNP dysfunction in a neurodevelopmental disease [21].

The assembly of box C/D snoRNPs is tightly regulated, and requires a set of dedicated *trans*-acting assembly factors that are conserved from yeast to humans [6]. This process is further coordinated by the help of chaperones including the HSP90-R2TP complex [22]. A critical box C/D snoRNP assembly factor, ZNHIT3 (zinc finger HIT-type containing protein 3; Hit1 in yeast) participates in a network of protein–protein interactions, forming a stable complex with the scaffolding protein NUFIP1 (Rsa1 in yeast) [23–25]. Upon inhibition of mTORC1, NUFIP1 is shown to act as an autophagy receptor to mediate the selective degradation of ribosomes via ribophagy [26]. Both in human and yeast, ZNHIT3/Hit1 is required to maintain the steady-state levels of its binding partner, NUFIP1/Rsa1 [23]. Further, proteomic analyses have shown that ZNHIT3 is part of an early protein-only pre-snoRNP assembly intermediate and is released upon binding of box C/D snoRNAs [25].

Deletion of the *HIT1* gene in yeast results in reduced steady-state levels of box C/D snoRNAs and impairs snoRNP assembly and ribosome biogenesis [23]. Studies in mouse models have recently reinforced the central role of ZNHIT3 in ribosome biogenesis and revealed that its disruption leads to downstream defects in translation during early embryonic development, thereby impairing cell lineage specification [27]. Consistent with these findings, ZNHIT3 deficiency in mice has also been linked to activation of p53/p21 signaling, and defects in cerebellar granule cell development, linking impaired ribosome biogenesis and neurodevelopmental phenotypes [28].

In humans, pathogenic variants of the *ZNHIT3* gene have been identified as the underlying cause of a severe neurodevelopmental condition known as PEHO syndrome, characterized by postnatal progressive cerebellar atrophy, edema, hypsarrhythmia, optic atrophy, arrest of psychomotor development, and a poor prognosis [29–31]. Heterozygous variants in *ZNHIT3* may also be associated with Mayer–Rokitansky–Küster–Hauser (MRKH) [32]. *ZNHIT3* has also been identified in transcriptomic studies as part of a prognostic gene signature in high-risk neuroblastoma [33], and as an independent predictor of disease progression in metastatic prostate cancer [34], further supporting its broader clinical relevance across human disease.

Only two PEHO-linked missense *ZNHIT3* variants have been described so far, both of which are located within the Zf-HIT domain of the protein. The founder Ser31Leu (S31L) variant was consistently identified as the cause of autosomal recessive PEHO syndrome in a cohort of Finnish descent. The second variant, Cys14Phe (C14F), was identified in one patient in the compound heterozygous state along with the S31L variant [31, 35]. Studies in human cell culture have shown that the *ZNHIT3*-S31L variation destabilizes the protein, decreasing steady-state protein levels [31]. Studies of these variants modeled in budding yeast demonstrated that pathogenic missense variants identified in individuals with PEHO syndrome reduce the steady-state levels of Hit1 protein. This decrease in protein levels is accompanied by a notable reduction in the cellular amounts of box C/D snoRNAs, site-specific rRNA modification changes, and impairment of rRNA processing and cellular translation [36].

Here, we report a severe fetal presentation associated with two novel *ZNHIT3* variants in the compound heterozygous state, characterized by hydrops fetalis and fetal lethality. Using genetic, biochemical, and cellular approaches, we demonstrate how these newly identified variants disrupt *ZNHIT3* function, impair snoRNP assembly, alter snoRNA abundance and rRNA modification, and ultimately compromise ribosome biogenesis and translation.

## Materials and methods

### Patient and clinical information collection

The study was conducted in accordance with the principles of the Declaration of Helsinki. As this noninterventional study analyzed fetal tissues obtained during clinical autopsies and involved no research procedures on living participants, approval by a research ethics committee was not required under the applicable French regulations. Written parental consent was obtained for publication of the fetal photographs. Complete autopsies of both fetuses were performed with the informed consent of both parents, in accordance with French regulations and according to standardized protocols including radiographs, photographs, macroscopic, and histological examinations of all viscera. Fetal biometric data were assessed by first-trimester sonography crown-rump length measurement and confirmed at autopsy by the evaluation of the fetal biometry, and organ and skeletal maturations as described previously [37]. Tissue samples from the lungs were collected during the autopsy for cryopreservation and potential future extraction of DNA or RNA.

Brains were removed and fixed in 10% buffered formalin supplemented with 3 g/l of ZnSO_4_ for ~2–3 weeks. Brains were then cut into coronal sections of 0.5 cm of thickness for routine examination. Seven-micrometer-thick paraffin-embedded tissue sections were performed on Superfrost Plus slides (Thermo Scientific) and stained with hematoxylin and eosin. Brains of fetuses resulting from late miscarriages without cerebral anomalies at autopsy were selected as controls.

### Molecular analysis

Whole genome sequencing analysis was performed in quartet on DNA extracted from cryopreserved fetal lung, and peripheral blood samples from parents with their informed consent.

### Cell culture and reagents

Human embryonic Kidney (HEK 293T) cells were obtained from ATCC and confirmed to be negative for mycoplasma using the Southern Biotech Mycoplasma Detection Kit. HEK293T cells were propagated in Dulbecco’s modified Eagle’s medium (DMEM; Corning) supplemented with 10% fetal bovine serum (Corning) with 1% penicillin–streptomycin (Gibco). The cells were maintained at 37°C with 5% CO_2_ in a humidified incubator. For transfection, HEK293T cells were seeded in six-well tissue culture plates at a density of 2 × 10^5^ cells/well and transfected with plasmids (2 µg total plasmid per well) on the following day using the Lipofectamine 3000 (Thermo Fisher Scientific) or PEI MAX reagent (Polysciences).

Lymphoblastoid cell lines from parents were maintained in RPMI 1640 medium (Deutscher) supplemented with 10% fetal calf serum (Gibco), 1% penicillin–streptomycin (Gibco), 2 mM glutamine, and 1% HEPES (Eurobio Scientific). RNA from these cells was used for RiboMethSeq analysis.

### Generation of *ZNHIT3* variant and short hairpin (shRNA) plasmids

The HA-tagged pRK5-*ZNHIT3* plasmid [26] (Addgene #110 953) was modified by site-directed mutagenesis to construct four plasmids expressing hemagglutinin (HA)-tagged *ZNHIT3* variants (*ZNHIT3*-C14R, *ZNHIT3*-C14F, *ZNHIT3*-S31L, and *ZNHIT3*-∆251–254). To recapitulate the patient-derived frameshift variant, the *ZNHIT3*-Δ251–254 expression construct was generated by site-directed mutagenesis using the wild-type (WT) *ZNHIT3* plasmid as the template. Specifically, the four-nucleotide sequence (AAGA) spanning nucleotide positions 251–254 of the *ZNHIT3* coding sequence was deleted, resulting in a frameshift. The frameshift altered the downstream coding sequence and introduced a premature stop codon, resulting in a truncated ZNHIT3 protein composed of the first 83 amino acids of the native protein followed by seven aberrant amino acids generated by the frameshift before termination (Fig. 1C).

**Figure 1.**
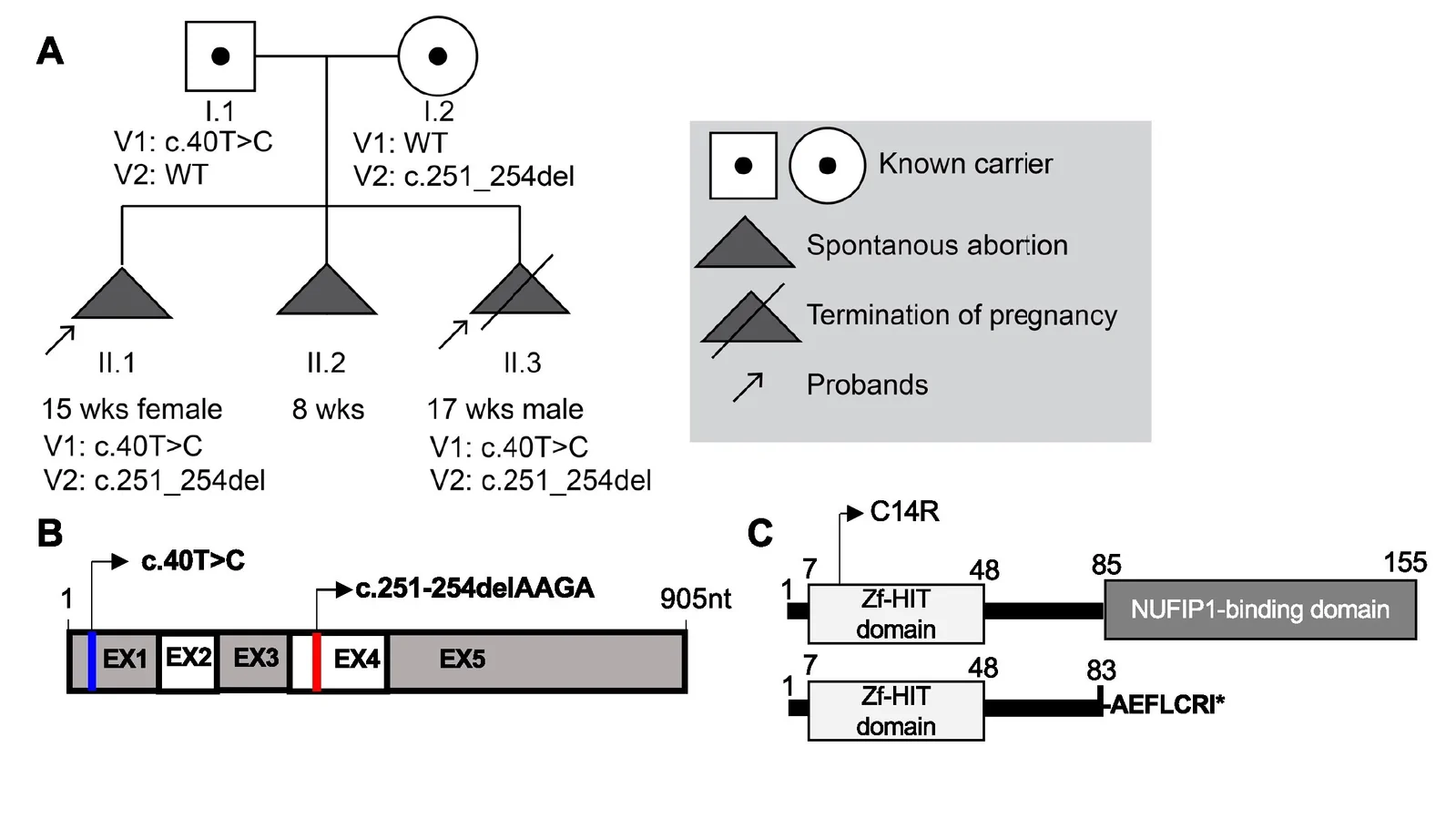
New pathogenic *ZNHIT3* variants causing fetal hydrops and lethality. (**A**) Pedigree of the family with two affected offspring. Shaded symbols indicate affected individuals. The genotype is shown for those individuals who were analyzed. All heterozygous carriers are phenotypically healthy. (**B**) Schematic of the location of the newly identified *ZNHIT3* variants. (**C**) Domain organization of ZNHIT3 protein and the newly identified variants.

The Lentiviral plasmids of *ZNHIT3*-WT, *ZNHIT3*-C14R, and *ZNHIT3*-∆251–254 were generated from the FUGW plasmid [38] (Addgene #14 883). To knockdown ZNHIT3 expression by shRNA, three shRNA oligonucleotides specific for *ZNHIT3* coding region were used. Oligonucleotides were annealed in a buffer containing 10 mM Tris, pH 7.6, and 20 mM NaCl by heating to 94°C for 3 min, followed by slow cooling to room temperature. Double-stranded oligonucleotides were then inserted into the plasmid vector pLVX-shRNA1 (NovoPro; V012697) via BamHI and EcoR1 restriction sites. As a negative control, a scrambled shRNA sequence was used. All plasmids used in this study were validated by whole plasmid sequencing and are listed in Supplementary Table S1. All oligos used in this study are listed in Supplementary Table S2.

### Lentivirus production and infection

Viruses were produced using FUGW-*ZNHIT3* plasmids transfected into HEK 293Tcells with polyethyleneimine (PEI 25K, Polyscience). In brief, cells were seeded at 3.8 × 10^6^ cells in a 10 cm tissue culture plates and incubated overnight at 5% CO_2_ at 37°C. Then, 1.3 pmol of psPAX2 (Addgene, #12 260), 0.72 pmol of pMD2.G (Addgene #12 259), and 1.64 pmol of FUGW-*ZNHIT3* plasmid were diluted in 500 μl Opti DMEM, then mixed with 500 μl DMEM with PEI (ratio 1 μg DNA: 3 μg PEI at 1 mg/ml). Virus within the complete medium was harvested at 48, 72 h post-transfection and centrifuged at 500 × *g* for 5 min to remove the packaging cells. The supernatant was filtered using a 0.45 μm polyethersulfone (PES) filter and then concentrated with a Lenti-X concentrator (TaKaRa #631 231). The prepared virus was aliquoted and stored at −80°C. The produced virus was validated through infection of HEK293T cells followed by immunoblotting to measure the ZNHIT3 protein level using the signal from the HA tag.

### Immunohistochemical staining

ZNHIT3 protein localization and expression in formalin-fixed paraffin-embedded brain tissues were performed with the OptiView DAB IHC Detection Kit (Ventana Medical Systems) on a BenchMark GX automated system (Ventana Medical Systems) using a rabbit anti-ZNHIT3 primary monoclonal antibody (1:50; HPA078211, Sigma–Aldrich). Signal intensity was enhanced using the OptiView Amplification Kit. Slides were digitized, and immunostaining intensities were compared using the ImageJ software.

### Immunoblotting and assay of protein stability

HEK293T cells were transfected with plasmids expressing HA-tagged WT or variant *ZNHIT3*. The cells were washed 48 h post-transfection with phosphate-buffered saline (PBS) and lysed in 250 µl of lysis buffer containing 150 mM NaCl, 20 mM Tris–HCl, pH 8.0, 1% NP-40, 10% sodium deoxycholate, and 10% sodium dodecyl sulphate–polyacrylamide gel electrophoresis (SDS). Total cell lysates were cleared by centrifugation [13 000 revolutions per minute (rpm), 15 min, 4°C], and the total protein concentration was measured using a Bicinchoninic Acid (BCA) protein assay kit (Pierce). Equal total protein concentrations were analyzed by sodium dodecyl sulphate–polyacrylamide gel electrophoresis (SDS–PAGE), followed by immunoblotting and quantification using Image Lab (Bio-Rad). To evaluate the stability of the *ZNHIT3* variants compared to the wildtype protein, transfected HEK293T cells were treated with 10 μg/ml cycloheximide (CHX) in DMEM at 37°C. Cells were harvested at time 0 and after 0.5, 1, and 2 h of incubation with CHX, washed with ice-cold PBS, and lysed in RIPA buffer. ZNHIT3 levels were measured by immunoblotting using an anti-HA antibody.

### Immunoprecipitation

For immunoprecipitation assays, transfected HEK293T cells were washed with ice-cold PBS and lysed in cold RIPA buffer containing protease inhibitors (Complete Mini, ethylenediaminetetraacetic acid-free; Roche). Lysates were kept on ice and clarified by centrifugation in a microcentrifuge at 13 000 rpm at 4°C for 10 min. The clarified lysate was incubated with anti-HA-tag magnetic beads with gentle rotation at 4°C for 90 min. The resin was washed three times with ice-cold lysis buffer. Bound samples were eluted with 0.1 M glycine at pH 2.5. Immunoprecipitated proteins were denatured by the addition of SDS loading buffer and boiling for 5 min and analyzed on SDS–PAGE followed by immunoblotting.

### Flow cytometry analysis of apoptosis and cell cycle

*ZNHIT3*-knockdown HEK293T cells were transiently transfected with plasmids encoding empty vector (EV), *ZNHIT3* or *ZNHIT3* variants using Lipofectamine 3000 (Thermo Fisher Scientific) according to the manufacturer’s instructions. Cells were harvested 48 h post-transfection for flow cytometry-based apoptosis and cell cycle analyses. For apoptosis assays, cells were washed twice with PBS and resuspended in 1 × Annexin V binding buffer. Cells were stained with PE-conjugated Annexin V and propidium iodide (PI) using the Annexin V apoptosis detection kit (BD Biosciences) following the manufacturer’s protocol. After incubation for 15 min at room temperature in the dark, samples were analyzed by flow cytometry. For cell cycle analysis, cells were washed with PBS, fixed in 70% ice-cold ethanol overnight at −20°C, and subsequently treated with RNase A (100 μg/ml) for 30 min at 37°C. Cells were stained with PI (50 μg/ml) for 30 min at room temperature in the dark. DNA content was measured by flow cytometry, and the proportions of cells in G0/G1, S, and G2/M phases were determined. Flow cytometry data were acquired on a NovoExpress flow cytometer and analyzed using FlowJo software (v10). Doublets were excluded by forward scatter height versus area gating. At least 10 000 events were collected per sample. All experiments were performed in at least three independent biological replicates.

### Total RNA extraction and Reverse Transcription Quantitative Polymerase Chain Reaction (RT-qPCR)

For fetal lung tissues and peripheral blood mononuclear cell (PBMC), total RNA was extracted with QIAGEN miRNeasy^®^ Mini kit according to the manufacturer’s instructions. For cell lines, total RNA was extracted with TRIzol (Invitrogen) and treated with DNase I (NEB) according to the manufacturer’s instructions. RNA concentration and purity were assessed using an Implen spectrophotometer (Implen Inc) using A260/A280 and A260/A230 absorbance ratios. The RNA was reverse transcribed with 50 ng random hexamers using Superscript III Reverse Transcriptase (Invitrogen) in a 20 μl reaction for 10 min at 25°C followed by 50 min at 50°C. No-reverse-transcriptase (no-RT) controls were included to assess amplification arising from residual genomic DNA. The complementary DNA (cDNA) was polymerase chain reaction (PCR) amplified using forward and reverse primers containing sequences specific to the snoRNAs (listed in Supplementary Table S2). In a 20 μl reaction, 3 ng of cDNA template, 0.5 μM each of forward and reverse primers, 10 μl Power-up Universal SYBR Green 2× Supermix (Applied biosystem) were mixed with nuclease-free water and amplified for 40 rounds at an annealing temperature of 60°C on a StepOnePlus Real-Time PCR System (Applied Biosystems). Primer amplification efficiencies were determined from standard curves generated using serial dilutions of cDNA. Amplification specificity was evaluated by melting-curve analysis, with a single predominant melting peak supporting specific amplification no-template controls were included for each primer pair and showed no specific amplification. GAPDH was used as the reference transcript for normalization. The suitability of GAPDH as a reference was evaluated by comparing its Ct values across the experimental groups, and no significant variation was observed among the conditions. Relative snoRNA expression was calculated using the 2^−ΔΔCt^ method. RT-qPCR experiments were performed using at least three independent biological replicates, with each biological sample analyzed in triplicate technical reactions. Technical replicate Ct values were averaged before calculation of relative expression, and statistical analyses were performed using values derived from independent biological replicates.

### RiboMethSeq analysis of rRNA modifications

The RiboMethSeq analysis, which measures 2′-O-methyl-induced protection of the RNA phosphodiester bond against alkaline hydrolysis, was performed as previously described, using the validated list of Nm positions present in *Homo sapiens* rRNA [39, 40]. Briefly, 100 ng of total RNA isolated from lung tissue samples from the second affected fetus and two age-matched controls, as well as from parental PBMC RNA, were subjected to alkaline hydrolysis at 96°C for 12 min. The precipitated and end-repaired RNA was then converted into a library using the NEBNext^©^ Small RNA Library Kit (NEB). The quality and quantity of the library was assessed using a High Sensitivity DNA Chip on a Bioanalyzer 2100 and a Qubit 2.0 fluorometer. High-throughput sequencing of the multiplexed libraries was performed on an Illumina NextSeq 2000 instrument in a 50 nt single end read mode. Raw modification scores are listed in Supplementary Table S3.

### Analysis of global translation by puromycin incorporation

Translation capacity was measured by quantifying the newly synthesized peptides using a puromycin incorporation assay in HEK293T cells transfected with wildtype or *ZNHIT3* variants. Puromycin was added to the media 48 h post-transfection to a final concentration of 10 μM, and cells were incubated for 60 min at 37°C. The cells were washed with ice-cold PBS and lysed in RIPA buffer. Lysed samples were clarified by centrifugation at 13 000 rpm for 10 min. Clarified samples were analyzed by western blotting. Anti-puromycin antibody was used to measure the amount of puromycin incorporation.

### Northern blot analysis

Total RNAs from three biological replicates were separated on a 1% agarose/formaldehyde gel and passively transferred to a Hi-bond nylon membrane (Cytiva). Membranes were probed with the indicated oligos. Signals were acquired with a Typhoon FLA 7000 (GE Healthcare) and bands were quantified using Image Lab software (Bio-Rad). Probe sequences are listed in Supplementary Table S1.

### Antibodies

All antibodies used in this study were commercially available and include HA tag monoclonal antibody (Invitrogen; 26 183), GAPDH monoclonal antibody (Proteintech; 60004-1-Ig), ZNHIT3 Polyclonal Antibody (Invitrogen; PA5-43577), ZNHIT3 Polyclonal Antibody (HPA078211, Sigma–Aldrich), Cyclophilin A Polyclonal Antibody (AF3589, R&D Systems), PIH1D1 Polyclonal Antibody (Proteintech; 19427-1-AP), RPAP3 Polyclonal Antibody (Proteintech; 23741-1-AP), NOP58 Polyclonal Antibody (Proteintech; 14409-1-AP), NUFIP1 Polyclonal Antibody (Proteintech; 12515-1-AP), NHP2L1 Polyclonal Antibody (Proteintech; 15802-1-AP), and anti-puromycin antibody (Sigma–Aldrich, MABE343).

### Statistical analysis

Statistical analyses were performed using GraphPad Prism (GraphPad Software). Data are presented as mean ± standard deviation (SD) from independent biological replicates. For experiments comparing *ZNHIT3*-knockdown cells complemented with EV, WT *ZNHIT3, ZNHIT3-*C14R, or *ZNHIT3-*Δ251–254, statistical significance was assessed using ordinary one-way analysis of variance (ANOVA) followed by Dunnett’s multiple-comparisons test, with WT *ZNHIT3* designated as the reference group. Exact sample sizes are provided in the corresponding figure legends. A *P*-value <.05 was considered statistically significant.

## Results

### Clinical findings and identification of novel pathogenic *ZNHIT3* variants

Healthy, unrelated 27-year-old parents were referred to the genetic consultation for multiple fetal losses. During the first pregnancy, fetal demise due to fetal hydrops occurred at 15 weeks of gestation (GW). Fetal pathology revealed a female fetus with normal growth parameters and no malformations but showed signs of placental hydrops (Supplementary Fig. S1A). Neuropathology examination was limited. Chromosomal anomaly investigations were negative. In the second pregnancy, an early miscarriage occurred at 8 GW for which no fetal examination was performed. The third pregnancy experienced a recurrence of fetal hydrops. The first-trimester ultrasound was normal, with a nuchal translucency of 1.7 mm and a crown-rump length of 60.9 mm. However, a morphologic ultrasound performed at 15 + 3 GW showed generalized edema and pericardial effusion. The medical termination of pregnancy took place at 17 GW. An autopsy revealed a slightly edematous male fetus with predominant edema in the extremities and slight retrognathia but no visceral malformation. Neuropathological examination showed measurements and maturation consistent with gestational age, without microscopic anomaly. Supplementary Figs S1B–D show the male fetus from the third pregnancy and the slightly edematous appearance of the face and limbs, with more pronounced edema at the extremities and tapered fingers. Supplementary Figs S1E and F show the macroscopic view of the brain after fixation for this fetus, compared to a normal control fetus of the same age (17 GW) shown in Supplementary Figs S1G and H. Microarray analysis and investigations for lysosomal storage diseases in the amniotic fluid sample were normal.

Whole-genome sequencing was performed on the fetuses from the first and third pregnancies and the parents. Two variants in the *ZNHIT3* gene were identified in both fetuses, each inherited from one parent (Fig. 1A). The paternal variant NM_004773.4: c.40T > C, p.(Cys14Arg), located in exon 1, is reported at extremely low frequency in population control databases (gnomAD v4). All *in silico* prediction tools tested (REVEL, Polyphen 2, Alphamissense) supported its deleterious nature (CADD score: 27.9). Moreover, another substitution affecting the same amino acid has been reported previously in a patient with PEHO syndrome [35]. The maternal variant NM_004773.4: c.251_254delAAGA, located in exon 4, is a frameshift deletion of 4 nucleotides predicted to lead to a premature stop codon [p.(Glu84Alafs*8)] reported with a frequency compatible with recessive transmission in population control databases (gnomAD v4) (Fig. 1B). Notably, no homozygous carriers of either the c.40T > C (p.Cys14Arg) or c.251_254del (p.Glu84Alafs8) variant have been reported in the gnomAD database. Thus, we identified two previously unreported *ZNHIT3* variants (Fig. 1C), which are distinct from previously reported *ZNHIT3* variants in their pathogenic manifestation and prompted investigation of their molecular and cellular consequences.

### *ZNHIT3* variants do not alter brain organization but differentially affect protein abundance and stability

To investigate the impact of the newly identified variants in the developing brain, we compared ZNHIT3 expression in brain tissue from one affected fetus and two age-matched control fetuses (~17 GW) by immunohistochemistry. The antibody used for these studies recognizes an N-terminal epitope of ZNHIT3 present in both variant proteins (Fig. 1C). Histological examination revealed no obvious abnormalities in cortical organization, neuronal migration, or cerebellar foliation at this developmental stage (Supplementary Figs S2 and S3). Similarly, the overall pattern of ZNHIT3 expression was comparable between affected and control tissues, with prominent staining observed in neuronal populations and germinal zones of the developing brain (Supplementary Fig. S2). These findings indicate that the two newly identified variants do not cause major defects in brain architecture or cause detectable ZNHIT3 expression changes during mid-gestation, suggesting that their consequences arise from molecular defects that are not apparent by histological analysis. We therefore next examined the effects of the variants on ZNHIT3 protein abundance and stability in a controlled cellular system.

Previously identified PEHO-associated *ZNHIT3* variants reduce steady-state protein levels in both yeast and human cells [31, 36]. To determine whether the newly identified *ZNHIT3* variants similarly affect the protein, we expressed HA-tagged WT or variant *ZNHIT3* in HEK293T cells and assessed protein abundance and stability using CHX chase assays. The maternally inherited c.251_254delAAGA variant is predicted to introduce a premature stop codon (p.Glu84Alafs*8) and remove the C-terminal region of ZNHIT3 containing the NUFIP1-binding domain. To investigate the functional consequences of disrupting this region, we generated a truncation construct lacking the corresponding C-terminal segment (hereafter referred to as the Δ251–254 model variant) and compared its properties with WT ZNHIT3 and the C14R variant. This construct was designed to model loss of the C-terminal region required for NUFIP1 interaction rather than the precise mutant transcript generated by the patient allele. Protein levels were monitored at 0, 0.5, 1, and 2 h following translation inhibition by immunoblotting with an anti-HA antibody and normalized to total protein per lane. The C14R variant clearly reduced steady-state ZNHIT3 levels relative to WT, whereas the Δ251–254 variant showed an increased protein abundance (Fig. 2A and B). Consistent with published reports, previously described PEHO-associated variants also showed reduced steady-state levels compared with WT (Fig. 2C and D).

**Figure 2.**
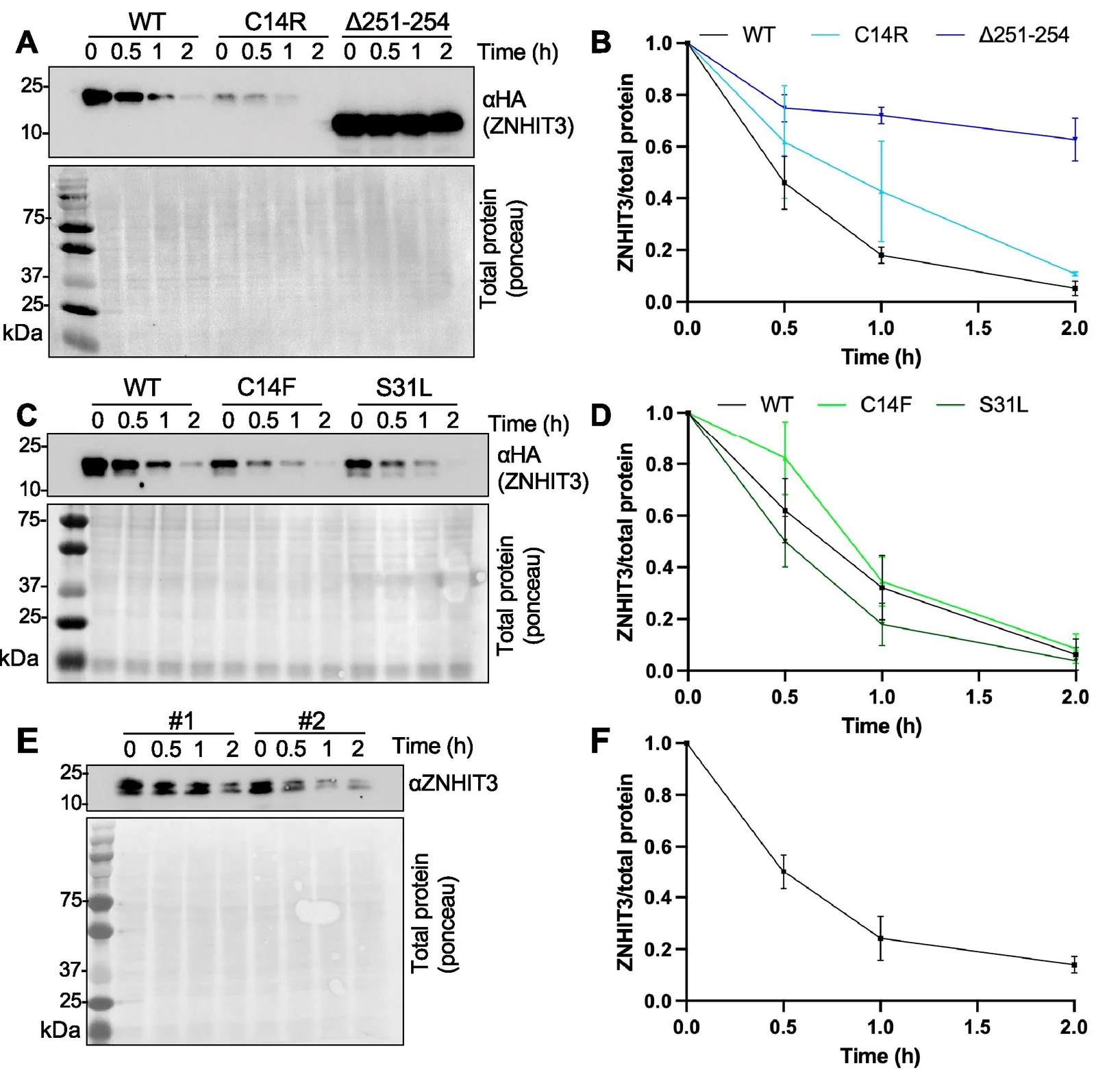
ZNHIT3 variants differentially impact protein abundance and stability in cells. (**A**) Representative immunoblots showing HA-tagged ZNHIT3 (WT and indicated variants) expressed in HEK293T cells treated with CHX for the indicated times (0, 0.5, 1, and 2 h) and detected with anti-HA antibody. Total protein staining is shown as a loading control. (**B**) Quantification of ZNHIT3 protein levels from CHX chase assays in panel (A), normalized to total protein and plotted relative to time 0. Data are presented as mean ± SD from three independent experiments. (**C**) Immunoblots of previously reported ZNHIT3 variants analyzed by CHX chase as in panel (A), with corresponding total protein controls. (**D**) Quantification of protein decay kinetics for variants shown in panel (C), normalized to total protein and expressed relative to time 0. Data show mean ± SD. (**E**) Representative immunoblot showing steady-state levels of endogenous ZNHIT3 in HEK293T cells, with total protein staining as a loading control. (**F**) Quantification of endogenously expressed ZNHIT3 protein levels from CHX chase assays in panel (E), normalized to total protein and plotted relative to time 0. Data are presented as mean ± SD from two independent experiments.

To determine whether these differences reflect changes in protein stability, we compared protein decay rates with endogenous ZNHIT3. Endogenous and HA-tagged WT ZNHIT3 exhibited similar half-lives (Fig. 2E and F), indicating that the HA tag and expression system do not substantially affect protein turnover. The Δ251–254 variant displayed increased stability relative to WT, consistent with its elevated steady-state abundance, whereas the remaining variants showed decay rates similar to WT despite reduced protein levels (Fig. 2A–D). Together, these results demonstrate that the two newly identified *ZNHIT3* variants have distinct effects on protein homeostasis. Whereas C14R reduces steady-state protein abundance, the Δ251–254 variant is more stable, indicating that *ZNHIT3* variants can perturb function through mechanisms other than protein destabilization.

### *ZNHIT3* variants do not alter subcellular localization but impair cell growth

Given that changes in subcellular localization can affect protein stability and function, we examined whether the *ZNHIT3* variants may affect intracellular distribution of the protein. Consistent with previous reports regarding localization of PEHO-associated ZNHIT3 variants [31], and our immunohistochemical analysis of patient tissue (Supplementary Fig. S2), WT ZNHIT3 and the newly identified variants were detected in both cytoplasmic and nuclear fractions of HEK293T cells (Fig. 3A). Fractionation quality was verified using GAPDH as a cytoplasmic marker, while endogenous ZNHIT6, another box C/D snoRNP biogenesis factor, served as a reference protein with a similar nucleocytoplasmic distribution. No substantial differences in localization were observed between WT and variant proteins, indicating that the effects of the variants on protein abundance and stability are unlikely to result from altered subcellular trafficking.

**Figure 3.**
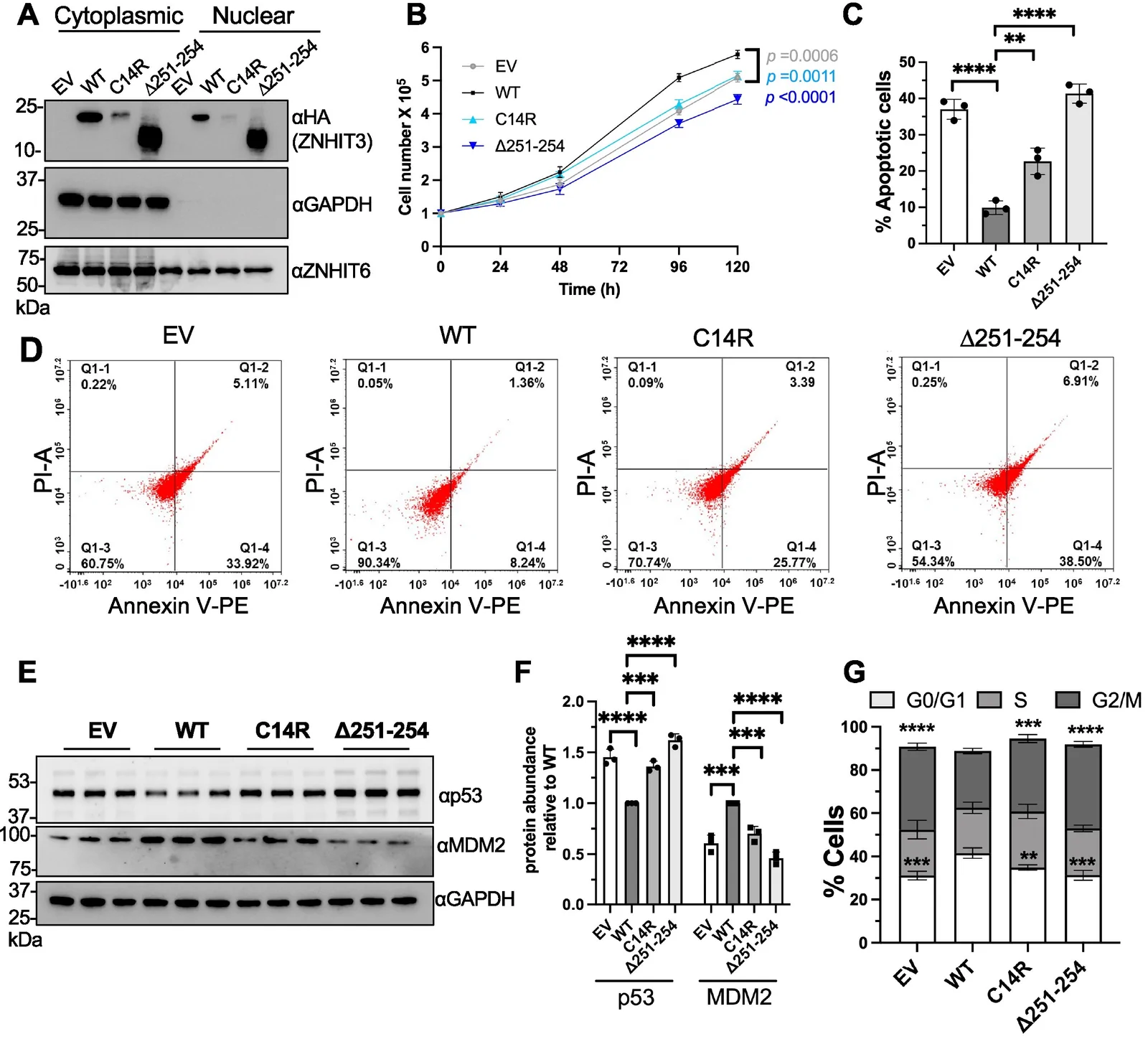
*ZNHIT3* variants impair cellular growth and activate stress-associated cellular responses. (**A**) Representative immunoblot showing expression of HA-tagged WT *ZNHIT3* and the indicated variants in *ZNHIT3* knockdown HEK293T cells after cellular fractionation. Cytoplasmic and nuclear extracts were prepared 48 h after transfection with *ZNHIT3* variants. GAPDH and ZNHIT6 are used as controls. (**B**) Growth analysis of *ZNHIT3*-knockdown HEK293T cells expressing EV, WT, C14R, or Δ251–254 *ZNHIT3*. Cell growth was monitored over 5 days and normalized to the initial measurement. Data represent mean ± SD from three independent biological replicates. *P*-values were determined for the final timepoint by two-way ANOVA with multiple-comparison correction. (**C**) Quantification of apoptotic cells from Annexin V/PI analysis shown in panel (D). (**D**) Representative flow cytometry plots of Annexin V-PE and PI staining in *ZNHIT3*-knockdown cells expressing EV, WT, C14R, or Δ251–254 ZNHIT3. (**E**) Immunoblots of p53 and MDM2 in *ZNHIT3*-knockdown HEK293T cells expressing EV, WT, C14R, or Δ251–254 ZNHIT3. GAPDH served as a loading control. (**F**) Quantification of p53 and MDM2 protein levels shown in panel (E), relative to GAPDH and normalized to WT ZNHIT3. (**G**) Cell cycle distribution determined by PI staining and flow cytometric analysis of *ZNHIT3*-knockdown HEK293T cells expressing EV, WT, C14R, or Δ251–254 ZNHIT3. Stacked bars indicate the percentage of cells in G1, S, and G2/M phases. Data in panels (C), (F), and (G) represent mean ± SD from three independent biological replicates. Statistical significance was determined using ordinary one-way analysis of variance (ANOVA) followed by Dunnett’s multiple-comparisons test. *P <.05, **P <.01, ***P <.001, and ^****^P <.0001.

To determine whether the variants impair ZNHIT3 function, we next assessed their ability to support cellular growth. Stable *ZNHIT3*-knockdown HEK293T cell lines were generated using two independent shRNAs, resulting in >80% depletion of ZNHIT3 at both the messenger RNA (mRNA) and protein levels (Supplementary Fig. S4A and B). We then expressed WT or variant *ZNHIT3* in knockdown cells and assessed growth over 5 days. The expression of WT *ZNHIT3* from the plasmid was comparable to expression of endogenous ZNHIT3 in HEK293T cells (Supplementary Fig. S4C). ZNHIT3 depletion significantly reduced cellular proliferation compared with WT-rescued cells, demonstrating that ZNHIT3 is required for normal cell growth (Fig. 3B). Expression of WT *ZNHIT3* in knockdown cells restored proliferation, whereas expression of neither variant rescued the growth defect. Cells expressing C14R showed a growth profile similar to knockdown controls and previously described PEHO variants, while the Δ251–254 variant produced an even more pronounced reduction in proliferation (Fig. 3B and Supplementary Fig. S4C). These findings indicate that pathogenic *ZNHIT3* variants impair cellular fitness through distinct mechanisms, as C14R and Δ251–254 have opposite effects on protein abundance yet both compromise cell growth. The stronger growth phenotype of the Δ251–254 variant, despite its increased stability, suggests that loss of specific molecular functions rather than reduced protein abundance alone underlies its pathogenicity.

### *ZNHIT3* variants induce apoptosis and alter cell cycle progression

Because impaired proliferation is frequently associated with activation of cellular stress responses, we next examined whether disruption of *ZNHIT3* affects apoptosis and cell cycle progression. Annexin V/PI staining revealed that knockdown of *ZNHIT3* significantly increased apoptosis, and this phenotype was rescued by expression of WT *ZNHIT3* (Fig. 3C and D). Expression of the C14R variant resulted in a modest increase in apoptosis relative to WT rescue, whereas the Δ251–254 variant induced a substantially stronger apoptotic response, consistent with its more severe growth defect. Given the established link between ribosome biogenesis defects and p53 signaling [41, 42], we next assessed components of the p53 pathway. Expression of WT *ZNHIT3* reduced p53 abundance relative to knockdown control cells, whereas expression of the Δ251–254 variant resulted in an increase in the steady-state p53 levels and the C14R variant produced a more modest effect (Fig. 3E and F). MDM2 levels were reduced in cells expressing the Δ251–254 variant; however, no significant consistent changes in p21 abundance were observed across conditions (Supplementary Fig. S4E).

We next investigated whether these changes were accompanied by changes in cell cycle progression. PI staining and flow cytometry revealed that *ZNHIT3* knockdown reduced the proportion of cells in G1 phase and increased the G2/M population relative to WT-rescued cells (Fig. 3G). Expression of WT *ZNHIT3* restored this distribution, whereas the Δ251–254 variant failed to rescue the defect and displayed a profile similar to knockdown control cells. Cells expressing the C14R variant showed an intermediate phenotype characterized by a modest decrease in G1 and increase in G2/M. Together, these results demonstrate that ZNHIT3 is required for normal cell survival and cell cycle progression. Furthermore, the two *ZNHIT3* pathogenic variants differentially impair these processes, with the Δ251–254 variant producing the strongest effects on apoptosis and cell cycle progression, consistent with its more severe impact on cellular fitness.

### The Δ251-254 variant disrupts interactions with snoRNP assembly factors

The Δ251–254 variant produced the most severe cellular phenotypes despite its increased protein stability, prompting us to investigate whether the pathogenic variants differentially affect the molecular functions of ZNHIT3 in box C/D snoRNP biogenesis. ZNHIT3 is a component of the snoRNP assembly machinery and interacts with assembly factors including NUFIP1 and PIH1D1. We therefore first examined whether expression of the pathogenic variants changes the abundance of proteins involved in this pathway. To assess the effects of the variants on snoRNP assembly factors, we expressed EV, HA-tagged WT, or variant ZNHIT3 in *ZNHIT3*-knockdown HEK293T cells, and the steady-state levels of key assembly factors and core snoRNP proteins were determined by immunoblotting. Expression of the C14R variant or EV reduced NUFIP1 abundance relative to WT-rescued cells, whereas cells expressing the Δ251–254 variant exhibited increased steady-state levels of NUFIP1 (Fig. 4A and B). PIH1D1 and 15.5K steady-state levels were also significantly reduced in cells expressing EV (Fig. 4A and B). In contrast, the steady-state levels of RPAP3, 15.5K, and Nop58 were not significantly changed in cells expressing either *ZNHIT3* variant.

**Figure 4.**
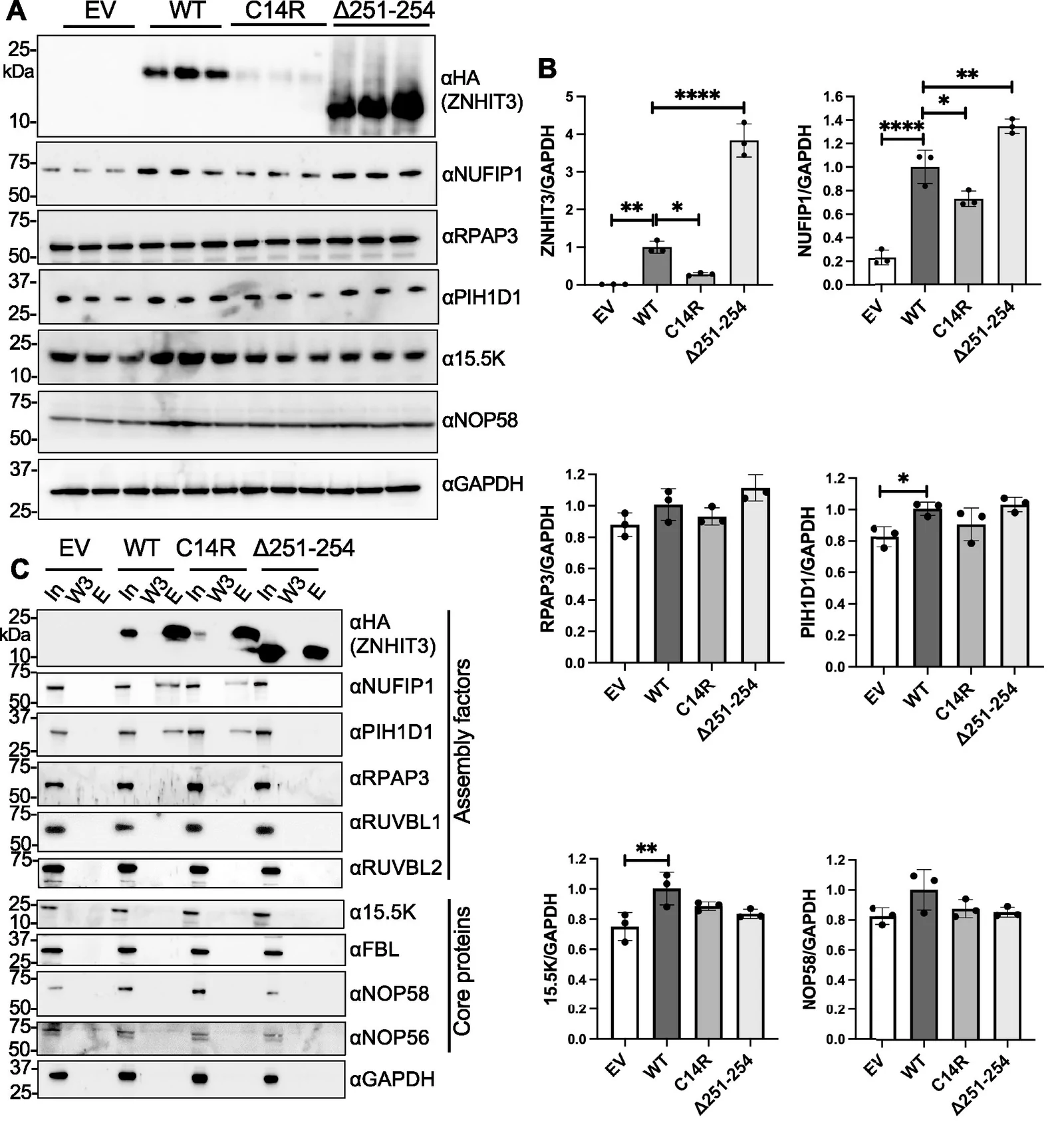
Pathogenic *ZNHIT3* variants alter snoRNP assembly factor abundance and disrupt interactions within the snoRNP assembly machinery. (**A**) Immunoblots showing steady-state levels of snoRNP assembly factors and core box C/D snoRNP proteins in *ZNHIT3*-knockdown HEK293T cells expressing EV, HA-tagged WT *ZNHIT3*, or the indicated *ZNHIT3* variants (C14R and Δ251–254). (**B**) Quantification of protein levels shown in panel (A). Protein abundance was normalized to GAPDH and expressed relative to WT ZNHIT3. Bars represent mean ± SD from three independent biological replicates. Statistical significance was determined using ordinary one-way analysis of variance (ANOVA) followed by Dunnett’s multiple-comparisons test. *P <.05, **P <.01, ***P <.001. (**C**) Co-immunoprecipitation analysis of HA-tagged WT ZNHIT3 and the indicated variants. Input lysates were normalized for total protein prior to pulldown. The difference in steady-state levels of variants in the input samples is consistent with their different stabilities. Recovery in the eluate fraction is constrained by the binding capacity of the affinity matrix. Associated proteins were detected by immunoblotting for snoRNP assembly factors and core snoRNP components. WT ZNHIT3 co-purified with NUFIP1 and PIH1D1, whereas the Δ251–254 variant failed to associate with either factor and the C14R variant showed reduced interaction. Similar amounts of HA-ZNHIT3 proteins were recovered in the eluate, enabling comparison of interaction efficiencies between variants. In, input; W3, final wash; E, eluate.

We next examined whether the newly identified variants affect assembly of ZNHIT3-containing complexes. HA-tagged WT or variant ZNHIT3 proteins were isolated by immunoprecipitation, and associated assembly factors and snoRNP proteins were analyzed by immunoblotting. Similar lysate concentrations were used, and comparable amounts of WT and variant ZNHIT3 proteins were recovered on beads, enabling direct comparison of interaction efficiencies (Fig. 4C). WT ZNHIT3 co-purified with both NUFIP1 and PIH1D1. In contrast, the Δ251–254 variant failed to co-purify either NUFIP1 or PIH1D1, consistent with deletion of the C-terminal region containing the NUFIP1-binding domain. The C14R variant retained interactions with both NUFIP1 and PIH1D1, although reduced amounts of each protein were recovered relative to WT ZNHIT3 (Fig. 4C).

Together, these results indicate that the two pathogenic variants impair ZNHIT3 function through distinct molecular mechanisms. Whereas the C14R variant primarily reduces ZNHIT3 abundance, the Δ251–254 variant is a stable protein that fails to associate with NUFIP1- and PIH1D1-containing complexes. Thus, in contrast to previously characterized pathogenic *ZNHIT3* variants that largely act through protein destabilization [36], the Δ251–254 variant disrupts the protein–protein interactions required for efficient box C/D snoRNP assembly. This difference provides a plausible explanation for the severe cellular defects observed despite increased protein stability.

### *ZNHIT3* variants reduce the abundance of specific box C/D snoRNAs

Because ZNHIT3 functions in box C/D snoRNP assembly and loss of ZNHIT3/Hit1 activity has previously been associated with reduced accumulation of box C/D snoRNAs [23, 36], we next examined whether the newly identified *ZNHIT3* variants affect snoRNA abundance. We quantified the steady-state levels of a targeted panel of box C/D snoRNAs in ZNHIT3-depleted HEK293T cells complemented with WT or variant *ZNHIT3* using RT-qPCR (Fig. 5A and B). The panel included box C/D snoRNAs representing diverse functional classes, including C/D snoRNAs guiding rRNA 2′-O-methylation, C/D snoRNAs involved in pre-rRNA processing, orphan box C/D snoRNAs, and, as controls, several box H/ACA snoRNAs.

**Figure 5.**
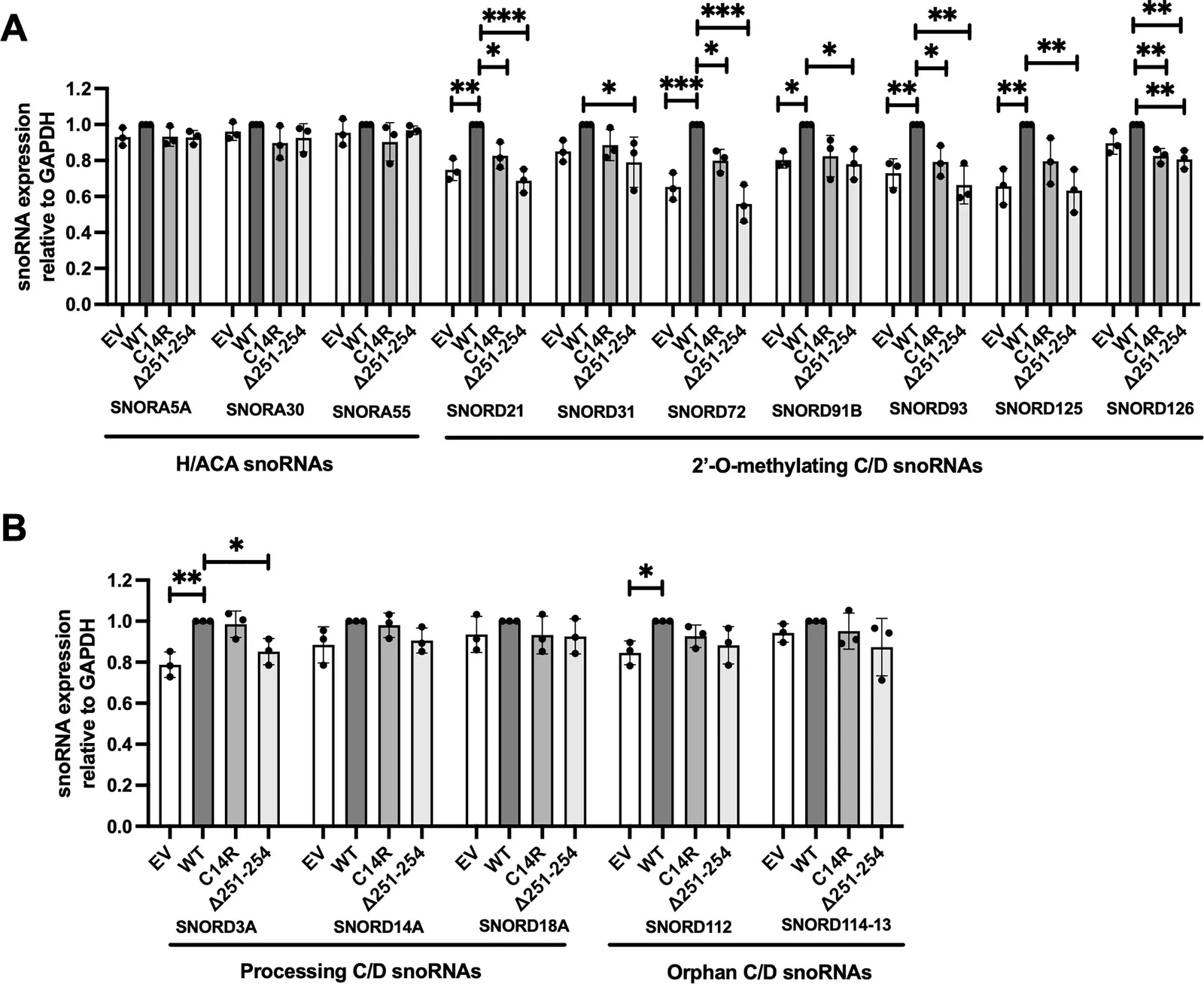
Pathogenic *ZNHIT3* variants reduce the abundance of specific box C/D snoRNAs. (**A**) RT-qPCR analysis of steady-state snoRNA levels in *ZNHIT3*-knockdown HEK293T cells expressing EV, WT *ZNHIT3*, or the indicated *ZNHIT3* variants (C14R and Δ251–254). The panel includes three H/ACA snoRNAs (SNORA5A, SNORA30, and SNORA55) and seven box C/D snoRNAs that guide rRNA 2′-O-methylation. snoRNA levels were normalized to GAPDH and shown relative to WT *ZNHIT3*. (**B**) RT-qPCR analysis of additional box C/D snoRNAs in *ZNHIT3*-knockdown HEK293T cells expressing EV, WT *ZNHIT3*, C14R, or Δ251–254 *ZNHIT3*. The panel includes box C/D snoRNAs involved in pre-rRNA processing (SNORD3A, SNORD14A, and SNORD18A) and orphan box C/D snoRNAs (SNORD112 and SNORD114-13). snoRNA levels were normalized to GAPDH and shown relative to WT *ZNHIT3*. In both panels (A) and (B), bars represent mean ± SD from independent biological replicates (*n* = 3). Statistical significance was determined using ordinary one-way analysis of variance (ANOVA) followed by Dunnett’s multiple-comparisons test. *P <.05, **P <.01, and ***P <.001.

Expression of either of the pathogenic *ZNHIT3* variants reduced the abundance of a subset of box C/D snoRNAs, whereas the tested box H/ACA snoRNAs were largely unaffected (Fig. 5A and B). Among the snoRNAs showing the most pronounced decreases were SNORD21, SNORD72, SNORD91B, SNORD93, SNORD125, and SNORD126. In contrast, several box C/D snoRNAs involved primarily in pre-rRNA processing, as well as all tested box H/ACA snoRNAs, showed little or no change relative to cells expressing WT *ZNHIT3*. Notably, the Δ251–254 variant generally produced stronger effects, consistent with the defect of this variant in binding key snoRNP assembly factors. Together, these data demonstrate that pathogenic *ZNHIT3* variants selectively impair the accumulation of a subset of box C/D snoRNAs. Given the established role of these snoRNAs in directing rRNA 2′-O-methylation, these findings prompted us to examine whether reduced snoRNA abundance is accompanied by altered rRNA modification patterns in patient tissues.

### *ZNHIT3* variants alter rRNA 2′-O-methylation at functionally important sites in patient tissue

To address whether the snoRNA accumulation defects associated with *ZNHIT3* variants result in altered rRNA modification patterns *in vivo*, we performed RiboMethSeq analysis on RNA isolated from preserved lung tissue of the affected fetus from the third pregnancy, two age-matched control fetuses, and parental PBMCs. Although PEHO syndrome primarily affects the nervous system, lung was the only frozen tissue available for RNA-based analyses following routine fetal autopsy. Given the ubiquitous requirement for box C/D snoRNP biogenesis and rRNA 2′-O-methylation, defects in these processes are expected to be detectable outside the central nervous system. RiboMethSeq analysis identified multiple hypomodified rRNA residues in the affected fetal sample relative to both fetal and parental controls (Fig. 6A). The most affected sites included 18S-Am576 and 18S-Cm1440 in the small ribosomal subunit and 28S-Gm1303, 28S-Am1310, 28S-Gm4166, and 28S-Um4590 in the large subunit. The complete rRNA 2′-O-methylation profiles are shown in Supplementary Fig. S6.

**Figure 6.**
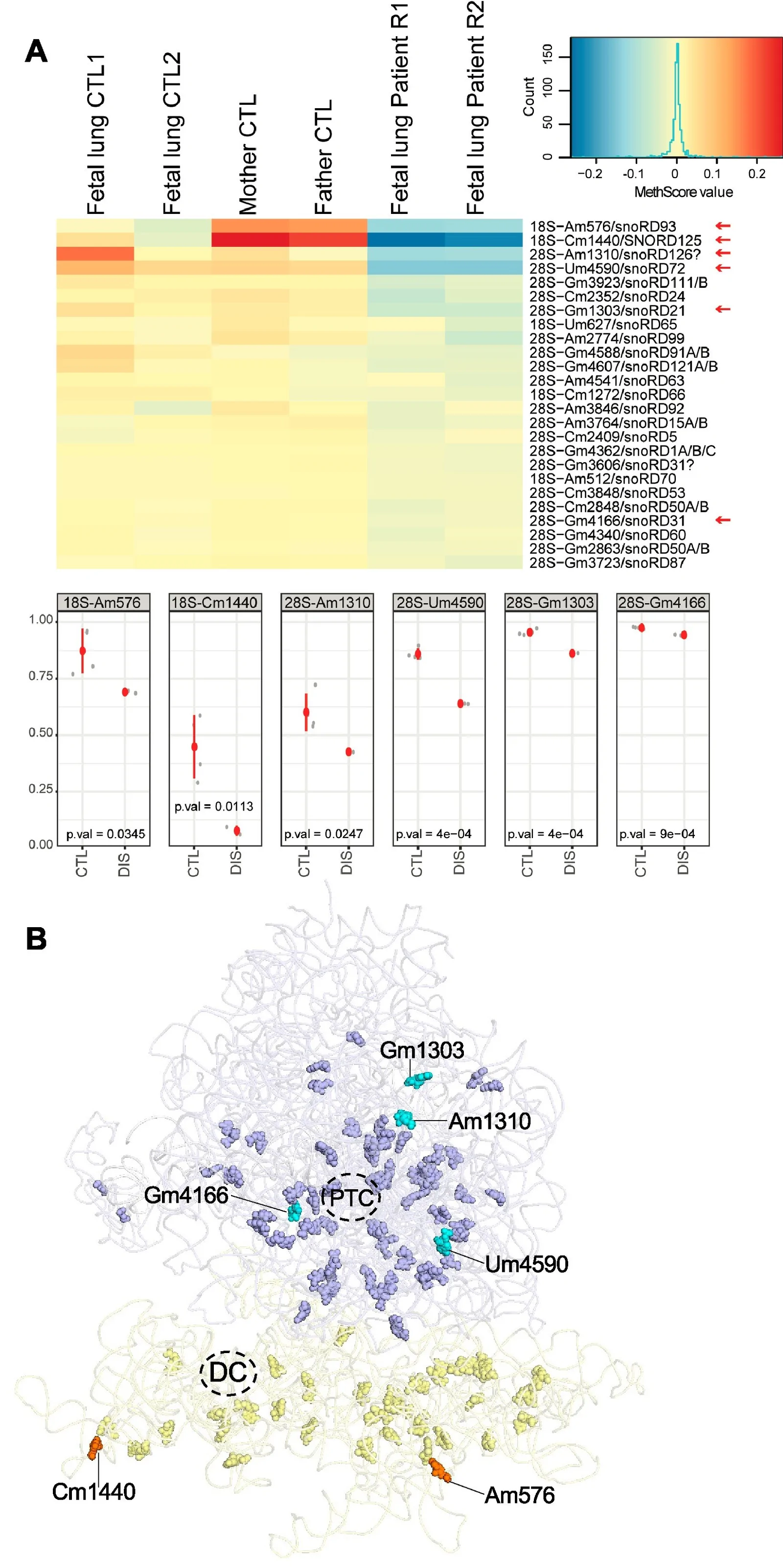
*ZNHIT3* variants impact rRNA 2′-O-methylation at specific sites in patient tissue. (**A**) RiboMethSeq analysis of rRNA 2′-O-methylation in RNA isolated from affected fetal lung tissue, age-matched fetal controls, and parental PBMC controls. Heatmaps show MethScores for selected 18S and 28S rRNA modification sites. Differentially modified sites are indicated (arrows). Representative MethScore profiles and quantification of selected sites comparing control (CTL) and affected disease (DIS) samples are shown below. Each gray dot represents measurement of an individual biological sample for each condition. Red dots indicate the group mean ± error SD; *P*-values are indicated. (**B**) Mapping of hypomodified rRNA residues onto the 3D structure of the human ribosome (PDB ID: 8QOI). Affected sites in the small ribosomal subunit (18S-Am576 and 18S-Cm1440; orange) and large ribosomal subunit (28S-Gm1303, 28S-Am1310, 28S-Gm4166, and 28S-Um4590; cyan) are highlighted. The positions of the decoding center and peptidyl transferase center (PTC) are indicated for reference. The affected modification sites are distributed across both ribosomal subunits and include several residues located within the functional core of the large subunit.

Several of the hypomodified residues are targets of box C/D snoRNAs whose abundance was reduced in cells expressing *ZNHIT3* variants (Fig. 5A), providing a direct link between defective snoRNP biogenesis and altered rRNA modification. To validate these findings independently, we examined the expression of two of these box C/D snoRNAs by northern blot analysis. Consistent with the RT-qPCR results, SNORD72 and SNORD125 were reduced in cells expressing the Δ251–254 variant compared with WT controls (Supplementary Fig. S5A and B). Notably, several of the affected modification sites were only partially methylated in control samples and are among the variably modified sites identified in other studies [43], indicating that these positions are intrinsically modified with lower efficiency and may therefore be particularly sensitive to perturbations in snoRNP biogenesis. Consistent with previous studies, the magnitude of 2′-O-methylation defects did not uniformly correlate with changes in guide snoRNA abundance, suggesting that modification efficiency is influenced by factors beyond snoRNA steady-state levels [12, 13, 44–46].

To examine the structural context of the affected modification sites, we mapped the hypomethylated residues onto the 3D structure of the human ribosome [47] (Fig. 6B). The affected modification sites were distributed across both ribosomal subunits rather than clustering within a single region. In the large subunit, 28S-Gm1303 and 28S-Am1310 form a conserved pair of neighboring modifications located near the PTC, while 28S-Gm4166 maps to the conserved Helix 69 region at the intersubunit interface and 28S-Um4590 localizes to the L1 stalk/E-site region involved in transfer RNA movement and ribosome dynamics. These findings indicate that pathogenic *ZNHIT3* variants affect modifications located in multiple functionally important regions of the ribosome.

We next compared the affected sites with published analyses of rRNA 2′-O-methylation variability and with our previous study of pathogenic variants in Hit1, the budding yeast ortholog of human *ZNHIT3* [36]. Of the six hypomethylated sites identified in patient tissues, five correspond to previously reported variable or partially modified 2′-O-methylation sites, whereas 28S-Gm4166 is considered a stable modification site [12, 43, 48, 49]. Notably, 28S-Gm4166 has an evolutionarily conserved yeast counterpart, 25S-Gm2619, which is generally considered a stable modification based on its limited variability across tested conditions. Interestingly, this yeast modification site was also found to be hypomethylated in budding yeast models of PEHO syndrome, caused by Hit1 dysfunction [36]. In contrast, another affected site identified in patient tissues, 18S-Cm1440, is among the most partially modified rRNA methylation sites reported across diverse human experimental systems.

Thus, the *ZNHIT3*-associated methylation defects identified here encompass both highly variable and stable rRNA modification sites, including the normally stable 28S-Gm4166. Together, these findings indicate that pathogenic *ZNHIT3* variants cause selective changes in rRNA 2′-O-methylation that at least for one conserved site overlap with those observed in the yeast Hit1 model. Notably, the affected sites span a broad spectrum of modification variability, ranging from highly variable sites such as 18S-Cm1440, to sites with low variability such as 28S-Am1310, and static sites such as 28S-Gm4166. These observations suggest that ZNHIT3 dysfunction compromises box C/D snoRNP activity sufficiently to affect both partially modified and normally stable rRNA methylation sites. Moreover, the conserved 28S-Gm4166/25S-Gm2619 modification was affected in both human patient tissue and budding yeast models, suggesting that this normally stable site may be especially susceptible to perturbations of the ZNHIT3/Hit1 pathway.

### The Δ251–254 variant impairs pre-rRNA maturation and ribosome biogenesis

Changes in the interaction network of ZNHIT3 variants with snoRNP assembly factors, reduced accumulation of specific box C/D snoRNAs, and site-specific hypo rRNA 2′-O-methylation patterns all could result in defects in ribosome biogenesis. To determine whether *ZNHIT3* variants affect pre-rRNA maturation, we analyzed rRNA processing by northern blotting using *ZNHIT3*-knockdown HEK293T cells expressing EV, WT, or ZNHIT3 variants. Equal amounts of total RNA from three independent biological replicates were analyzed with probes complementary to the 5′-ETS, ITS1, and ITS2 regions of the human pre-rRNA transcript (Fig. 7A) as well as probes for mature 18S, 28S, and 5.8S. We also probed the level of GAPDH transcript as a loading control.

**Figure 7.**
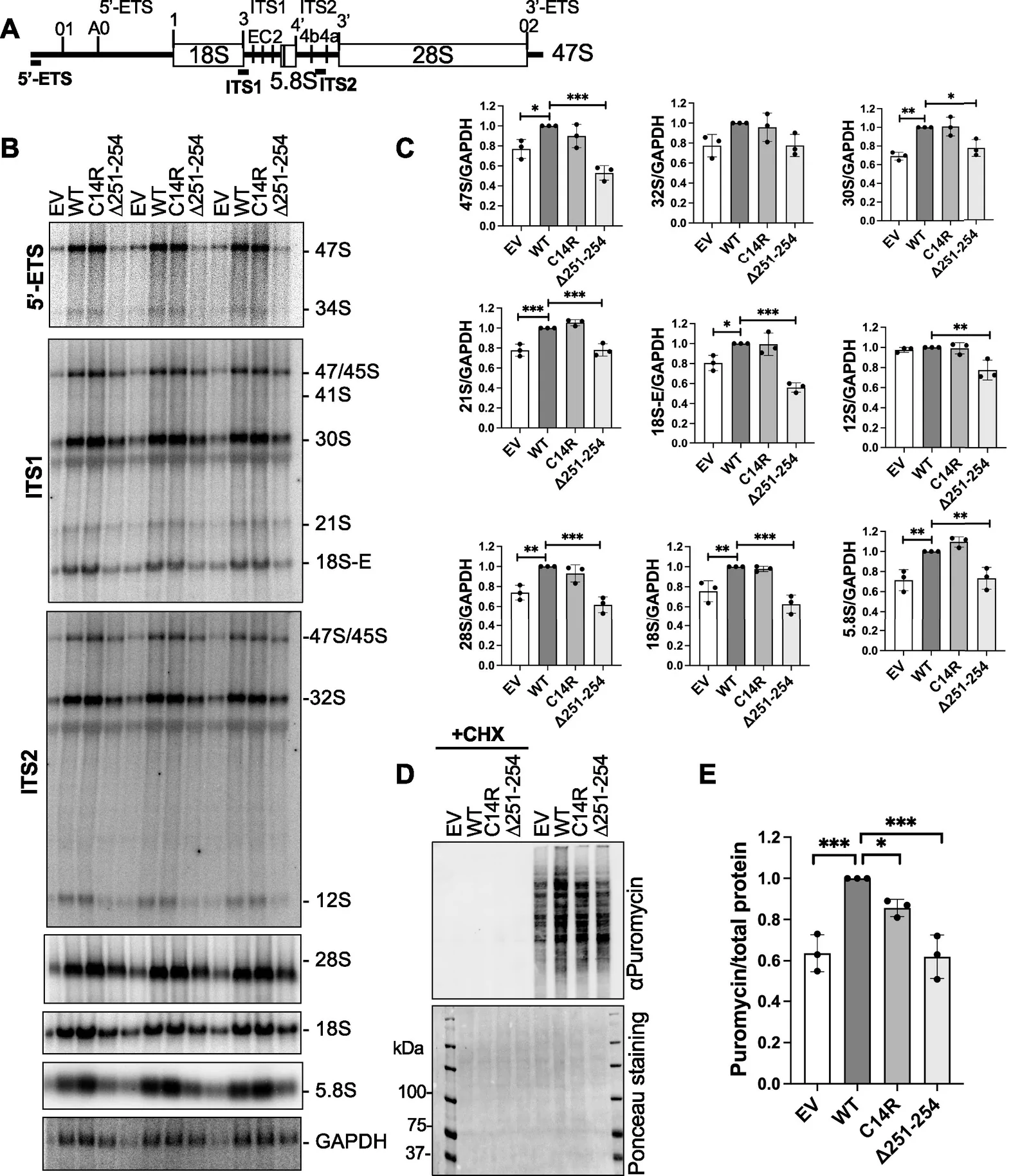
The Δ251–254 *ZNHIT3* variant impairs pre-rRNA maturation and reduces translational output. (**A**) Schematic of the human 47S pre-rRNA transcript showing the positions of the 5′-ETS, ITS1, and ITS2 probes used to detect pre-rRNA processing intermediates. (**B**) Representative northern blot analysis of pre-rRNA processing in *ZNHIT3*-knockdown HEK293T cells expressing EV, WT *ZNHIT3*, or the indicated *ZNHIT3* variants (C14R and Δ251–254). GAPDH mRNA was probed as a loading control. (**C**) Quantification of rRNA processing intermediates and mature rRNAs detected in panel (B). Signal intensities were normalized to GAPDH and are shown relative to WT *ZNHIT3*. Data represent mean ± SD from three independent biological replicates. Statistical significance was determined using unpaired two-tailed *t*-tests. *P <.05, **P <.01, ***P <.001. (**D**) Representative puromycin incorporation assay measuring global protein synthesis in *ZNHIT3*- knockdown HEK293T cells expressing EV, WT ZNHIT3, C14R, or Δ251–254. Newly synthesized proteins were detected by immunoblotting using an anti-puromycin antibody. CHX-treated cells served as a negative control. Ponceau staining is shown as a loading control. (**E**) Quantification of puromycin incorporation shown in panel (**D**), relative to total protein and normalized to WT *ZNHIT3*. Data represent mean ± SD from three independent biological replicates. Statistical significance was determined using ordinary one-way analysis of variance (ANOVA) followed by Dunnett’s multiple-comparisons test. *P <.05, **P <.01.

*ZNHIT3*-knockdown cells expressing EV showed a reduction of precursor rRNAs, including 47S, 30S, 21S, 18S-E, as well as mature rRNAs, consistent with early defects in ribosome biogenesis. Cells expressing WT *ZNHIT3* and the C14R variant showed similar levels of most pre-rRNA species, whereas expression of the Δ251–254 variant significantly reduced the abundance of 47S and 34S pre-rRNAs, which were detected by a probe binding to the 5′-ETS region, suggesting impaired accumulation of early pre-rRNA intermediates in these cells. Analysis of ITS1-containing intermediates revealed a broader defect associated with the Δ251–254 variant. Relative to WT control cells, expression of Δ251–254 significantly reduced the levels of the 30S, 21S, and 18S-E species, indicating impaired maturation of precursors required for small ribosomal subunit production (Fig. 7B and C). In contrast, expression of the C14R variant had no significant effect on the abundance of these intermediates. Cells expressing the Δ251–254 variant also accumulated significantly lower levels of the 12S pre-rRNAs compared with WT cells, whereas the processing profile of the C14R variant remained largely similar to WT (Fig. 7B and C). Consistent with these changes in pre-rRNA maturation, mature 18S, 28S, and 5.8S rRNA levels were reduced in cells expressing the Δ251–254 variant similar to *ZNHIT3*-knockdown cells. By contrast, the C14R variant produced no significant effect on mature rRNA abundance.

Together, these data demonstrate that the Δ251–254 variant causes a broad defect in ribosome biogenesis characterized by reduced accumulation of pre-rRNA intermediates involved in both small- and large-subunit maturation. In contrast, the C14R variant has little detectable impact on pre-rRNA processing despite reducing ZNHIT3 protein abundance. These findings further support the conclusion that the severe cellular phenotypes associated with Δ251–254 arise from disruption of critical molecular functions required for snoRNP assembly and ribosome production.

### *ZNHIT3* variants reduce translational output

The defects in pre-rRNA maturation observed in cells expressing the Δ251–254 variant suggested that pathogenic *ZNHIT3* variants are likely to ultimately compromise protein synthesis. To determine whether the molecular defects associated with *ZNHIT3* variants affect translation, we measured global protein synthesis using a puromycin incorporation assay. Newly synthesized proteins were pulse-labeled with puromycin and detected by immunoblotting using an anti-puromycin antibody (Fig. 7D and E). Expression of either pathogenic *ZNHIT3* variant significantly reduced puromycin incorporation relative to WT ZNHIT3, indicating decreased translational output (Fig. 7D and E). The most pronounced reduction was observed in cells expressing the Δ251–254 variant, consistent with its substantial effects on snoRNP assembly, rRNA modification, and pre-rRNA processing.

Notably, the C14R variant also reduced translation despite minor defects in pre-rRNA maturation. This finding suggests that translational output is particularly sensitive to reduced ZNHIT3 function and may be affected by defects that are not fully captured by steady-state analyses of ribosome biogenesis. Consistent with its reduced protein abundance, the C14R variant likely causes a partial loss of ZNHIT3 activity that is sufficient to impair protein synthesis without producing significant pre-rRNA processing defects. The convergence of defects in snoRNP assembly, snoRNA accumulation, rRNA modification, pre-rRNA maturation, and translation supports a critical role for ZNHIT3 in maintaining ribosome function and proteome homeostasis.

## Discussion

PEHO syndrome is a rare neurodevelopmental disorder that is most commonly associated with the Finnish founder variant p.Ser31Leu in *ZNHIT3*. Here, we expand both the clinical and molecular spectrum of *ZNHIT3*-associated disease by describing two non-Finnish siblings carrying compound heterozygous *ZNHIT3* variants, p.C14R and p.Δ251–254. To our knowledge, these represent the first reported prenatal cases associated with biallelic *ZNHIT3* variants, characterized by early hydrops fetalis, with one pregnancy resulting in intrauterine demise and the other undergoing medical termination following prenatal diagnosis. This unusually severe presentation broadens the phenotypic spectrum associated with ZNHIT3 deficiency and demonstrates that disruption of ZNHIT3 function can compromise human development well before the onset of the classical postnatal neurological manifestations of PEHO syndrome.

The developmental expression pattern of ZNHIT3 is likewise consistent with the clinical manifestations of PEHO syndrome. We observed strong expression in fetal germinal zones, brainstem structures, cerebellar pathways, corpus callosum, and optic tracts, corresponding closely to the anatomical regions most severely affected in PEHO syndrome. Although no major neuropathological abnormalities were apparent in the 15-week fetus examined in this study, this finding is compatible with the progressive nature of the neurological phenotype. Indeed, microcephaly typically develops during infancy, while cerebellar and optic atrophy become apparent during postnatal disease progression, suggesting that the developmental consequences of ZNHIT3 deficiency become most apparent during later stages of neurodevelopment when demands for ribosome production and protein synthesis are particularly high [31].

Although ZNHIT3 has long been recognized as a component of the box C/D snoRNP assembly machinery, the molecular consequences of disease-causing *ZNHIT3* variants have remained poorly understood. By comparing two novel disease-associated variants, our study demonstrates that pathogenic *ZNHIT3* variants impair cellular function through distinct mechanisms. Similar to the previously reported PEHO-associated variants C14F and S31L [31], the C14R variant primarily reduces ZNHIT3 protein abundance, further supporting protein destabilization as a recurrent pathogenic mechanism in ZNHIT3-associated disease. Notably, two independent disease-associated substitutions have now been identified at residue Cys14, suggesting that this N-terminal region is particularly sensitive to amino-acid substitutions that compromise ZNHIT3 stability. In contrast, in our ectopic expression system, the Δ251–254 allele produced a detectable and relatively stable truncated protein that was functionally compromised, thereby uncoupling protein abundance from activity. This variant exhibited substantially greater effects across molecular and cellular readouts, including defective interactions with snoRNP assembly factors, reduced accumulation of a subset of box C/D snoRNAs, altered rRNA modification patterns, impaired pre-rRNA maturation, reduced translational output, decreased proliferation, and increased apoptosis. These findings suggest that Δ251–254 behaves as a loss-of-function variant and identify disruption of snoRNP assembly factor interactions as a major molecular consequence of this variant. More broadly, our results define how specific disease variants of *ZNHIT3* perturb cellular function, providing an allele-specific framework for understanding phenotypic variability in *ZNHIT3*-associated disease. It should be noted that the Δ251–254 variant examined in this study was expressed from an ectopic expression construct and thus was used to assess the functional properties of the protein encoded by the mutant allele. Because the corresponding patient-derived c.251_254del variant introduces a premature termination codon, the endogenous mutant transcript may be subject to nonsense-mediated mRNA decay *in vivo*. Consequently, the pathogenic mechanism in patients may involve both loss of ZNHIT3 protein production due to reduced transcript abundance and impaired function of any mutant protein that escapes transcript surveillance. This distinction should be considered when interpreting the functional analyses presented here.

A notable observation is that pathogenic *ZNHIT3* variants did not uniformly affect all box C/D snoRNAs. Instead, only a subset of snoRNAs showed reduced steady-state abundance, indicating that individual snoRNAs differ in their dependence on efficient ZNHIT3-mediated assembly. Importantly, this phenomenon is not unique to our human cellular models. Previous studies in yeast demonstrated that disruption of snoRNP assembly pathway similarly affects only a subset of box C/D snoRNAs and rRNA modifications [36, 46], suggesting that selective sensitivity to snoRNP assembly defects is an evolutionarily conserved feature of the pathway. Selective effects on box C/D snoRNA abundance have also been reported in patient-derived fibroblasts carrying a pathogenic *NOP58* variant, where several box C/D snoRNAs that guide rRNA 2′-O-methylation were significantly reduced, whereas SNORD3A, a box C/D snoRNA involved in pre-rRNA processing, remained unaffected [21]. Thus, differential sensitivity among individual box C/D snoRNAs appears a recurring feature of snoRNP biogenesis related disorders, but the molecular basis for this selectivity remains unclear. Box C/D snoRNAs differ substantially in abundance, stability, intracellular trafficking, and interactions with both proteins and target RNAs [6, 50–53]. Consequently, individual snoRNAs may exhibit distinct requirements for efficient assembly and maturation. Our findings support a model in which ZNHIT3 deficiency preferentially destabilizes a vulnerable subset of snoRNAs rather than causing a global collapse of box C/D snoRNPs.

Importantly, the selective loss of snoRNAs was accompanied by measurable changes in rRNA modification patterns in some cases, demonstrating that relatively modest perturbations in snoRNA homeostasis can have downstream consequences. Most of the affected modification sites correspond to naturally variable modifications previously identified in human RiboMethSeq studies, whereas 28S-Gm4166 is generally considered as a highly stable modification site [49]. The affected sites therefore span a range of known sensitivities. Comparison with the budding yeast Hit1 model revealed overlap at a single conserved and highly stable modification site, suggesting that modification at 28S-Gm4166/ 25S-Gm2619 is particularly sensitive to ZNHIT3/Hit1 dysfunction [36]. The selective nature of rRNA modification defects may also have broader implications for disease pathogenesis. Increasing evidence suggests that rRNA modifications are not simply static structural features of ribosomes but can influence translational efficiency, fidelity, and responsiveness to cellular demands [12, 45, 46, 54–59]. In this context, pathogenic *ZNHIT3* variants may influence ribosome function both quantitatively, through reduced ribosome production, and qualitatively, through altered patterns of rRNA modification.

Our findings regarding the critical developmental role of ZNHIT3 are further strengthened by recent studies in conditional mouse models demonstrating that Znhit3 is essential for cerebellar granule cell development and maintenance of nucleolar homeostasis. Consistent with early observations linking ZNHIT3 deficiency to cerebellar granule neuron loss in PEHO syndrome, conditional ablation of Znhit3 in cerebellar granule cell lineages resulted in impaired granule cell progenitor survival, proliferation, differentiation, and migration, leading to progressive cerebellar hypoplasia and motor dysfunction [28, 31]. Defective rRNA processing and nucleolar stress were identified as central molecular consequences of Znhit3 loss, further supporting a critical role for ZNHIT3 in ribosome biogenesis during neurodevelopment. Importantly, these *in vivo* observations complement our molecular analyses of pathogenic *ZNHIT3* variants and support the conclusion that disruption of ribosome biogenesis likely represents the primary pathogenic consequence of ZNHIT3 deficiency.

We show that translational output is reduced not only in cells expressing the severe Δ251–254 variant but also in cells expressing C14R, despite the relatively modest effect of this variant on pre-rRNA processing. This finding is consistent with our observations in budding yeast model [36], and suggests that protein synthesis is particularly sensitive to partial ZNHIT3 deficiency and informs how subtle alterations in snoRNP assembly, rRNA modification, or ribosome composition can impair translation. These observations are consistent with emerging concepts in ribosomopathy biology, in which relatively modest perturbations in ribosome biogenesis can have profound biological consequences through both quantitative reductions in ribosome number and translational capacity, as well as qualitative changes in translation [60–62].

In conclusion, this study expands the clinical spectrum of PEHO syndrome to include antenatal disease associated with hydrops fetalis and fetal loss and provides mechanistic insight into how pathogenic *ZNHIT3* variants disrupt cellular homeostasis. Our findings support a model in which pathogenic *ZNHIT3* variants, regardless of their underlying molecular mechanism, compromise box C/D snoRNP assembly, alter snoRNA homeostasis and rRNA modification, and ultimately reduce translational output. Together with recent *in vivo* evidence [27, 28], these results establish defective ribosome biogenesis and translation as central pathogenic mechanisms underlying *ZNHIT3*-associated disease. More broadly, our work strengthens the growing link between defects in ribosome biogenesis and selective neurodevelopmental vulnerability, highlighting how perturbation of a fundamental and ubiquitous cellular pathway can result in highly specific developmental pathology.

## Supplementary Material

gkag1024_Supplemental_File

## Data Availability

RiboMethSeq data are deposited to the European Nucleotide Archive and are available under accession code PRJEB78345 (https://www.ebi.ac.uk/ena/browser/view/PRJEB78345).
