# Supplementary material for: New pathogenic ZNHIT3 variants causing fetal hydrops and lethality disrupt snoRNP assembly
Source: Nucleic Acids Res. 2026 Nov 5;54(20):gkag1024. doi: 10.1093/nar/gkag1024 (PMC13736010; doi:10.1093/nar/gkag1024)
Supplement: Supplementary file 1 [file gkag1024_supplemental_file.pdf]

New pathogenic ZNHIT3 variants causing prenatal PEHO syndrome disrupt snoRNP assembly

AUTHORS

AUTHORS

Md Lutfur Rahman[1,#], Adeline A. Bonnard[2,3,#,*], Lyse Ruaud[2,3], Fabien Guimiot[2,3], Ines Defer[2,3], Feng Wang[4], Virginie Marchand[5], Yuri Motorin[6], Bing Yao[4], Séverine Drunat[2,3] and Homa Ghalei[1,*]

[1]Department of Biochemistry, Emory University School of Medicine, Atlanta, 30322 Georgia, USA.

[2]Département de Génétique, Hôpital Robert Debré, Assistance Publique des Hôpitaux de Paris (AP-HP), 75019 Paris, France.

[3]INSERM UMR 1141, Paris-Cité University, NeuroDiderot, 75019 Paris, France.

[4]Department of Human Genetics, Emory University School of Medicine, Atlanta, 30322 Georgia, USA.

[5]Université de Lorraine, SMP IBSLor, Biopôle, 9 Avenue de la Forêt de Haye, 54505 Vandoeuvre-les-Nancy, France.

[6]Université de Lorraine, UMR7365 IMoPA, CNRS, Biopôle, 9 Avenue de la Forêt de Haye, 54505 Vandoeuvre-les-Nancy, France.

*To whom correspondence should be addressed: Adeline Bonnard and Homa Ghalei; Tel: +1 404-727-6800; Email: adelinealice.bonnard@aphp.fr and hghalei@emory.edu

#Joint Authors

Content warning: Supplementary Figure 1 contains images of deceased fetal specimens. Some readers may find these images distressing.

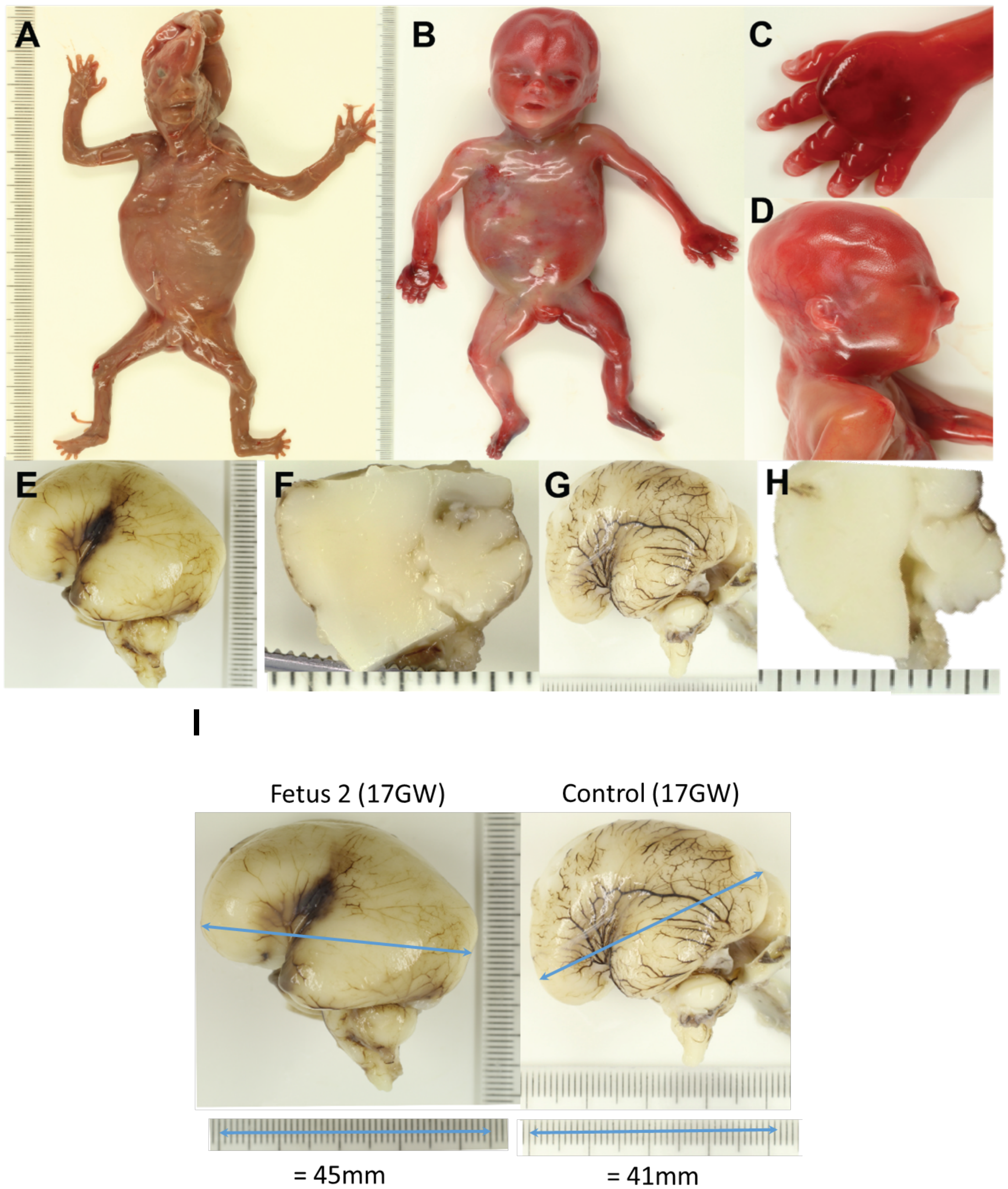

Figure S1. Macroscopic description of affected and control fetuses. (**A**) Female fetus from the first pregnancy presents highly macerated integument. (**B-D**) Features of the male fetus from the third pregnancy. The fetus shows a slightly edematous appearance of the face and limbs, with more pronounced edema at the extremities and tapered fingers. (**E-H**) Macroscopic view of the fetal brains after fixation: (**E**) Left lateral view of the affected male fetus brain, (**F**) the midsagittal section of the cerebellum of the affected male fetus. (**G-H**) Similar views of a normal control fetus of the same age (17 GW). (**I**) Fetal brain measurements. The two brains were enlarged to the same size by aligning the two scales. Measurements taken at autopsy for the left occipitofrontal diameter (blue double arrow) were 40 mm for both fetuses, placing them in the 25th percentile. Measurements taken from the photograph were 45 mm and 41 mm for the diseased and control fetuses, respectively.

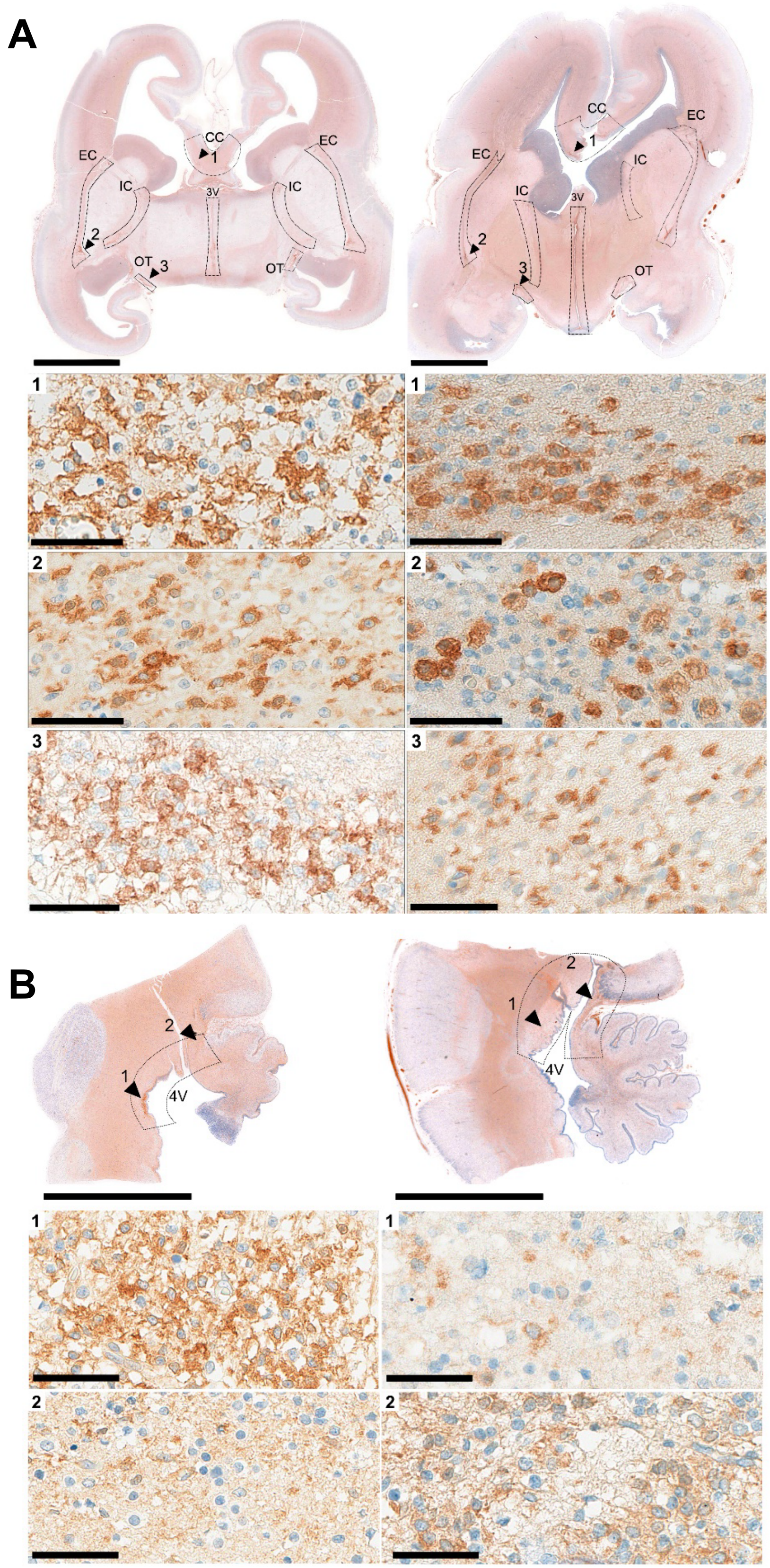
A
CC
1
EC
IC
3V
2
OT
3
B
4V

C

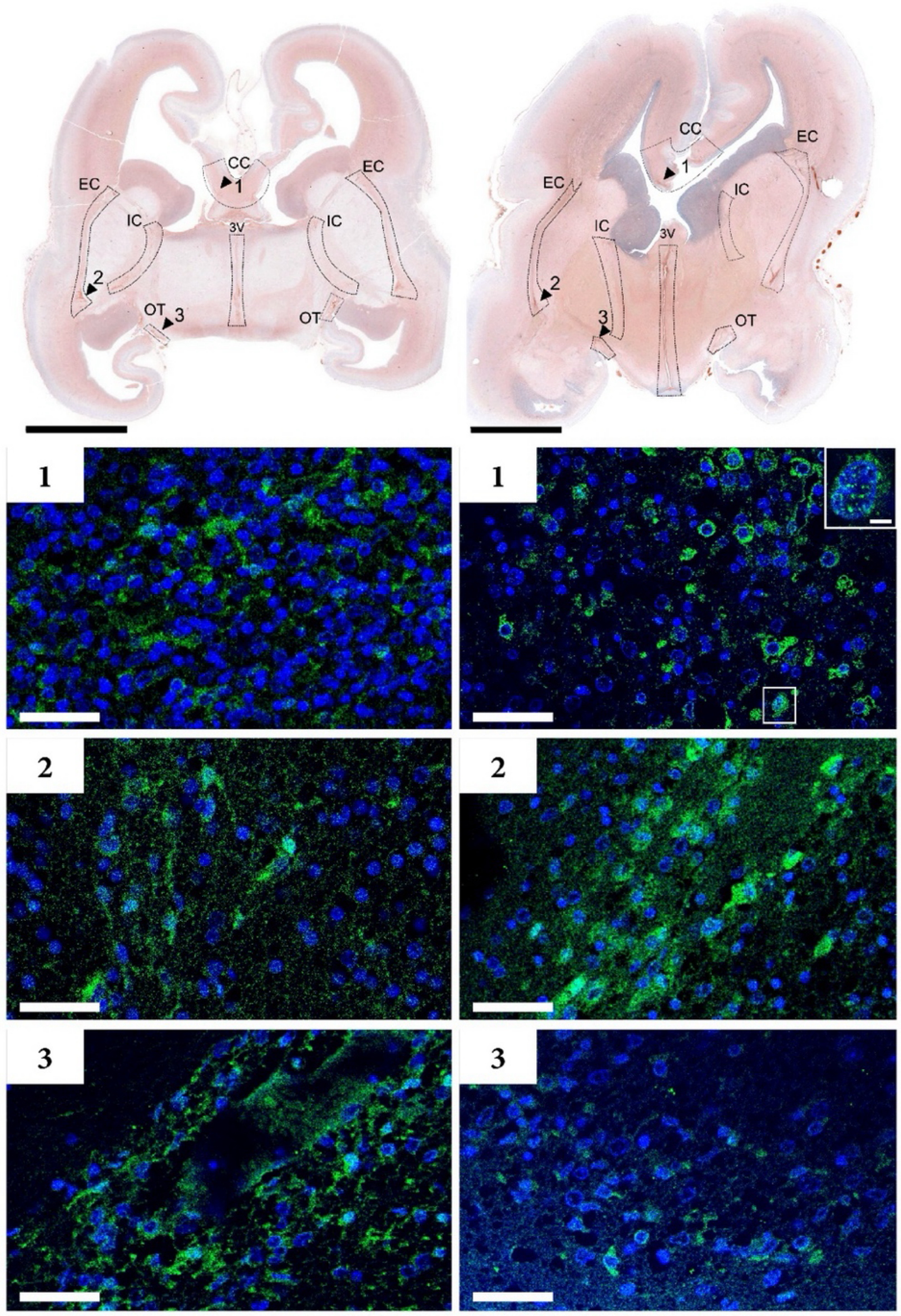

Figure S2. Analysis of ZNHIT3 expression in brain sections. ZNHIT3 expression was analyzed in the developing brain and cerebellum at 17 gestational weeks using immunohistochemistry on paraffin-embedded sections. The right panels display results from the affected male fetus, while the left panels show age-matched control samples. (**A**) DAB-stained coronal brain sections. (**B**) DAB-stained sagittal cerebellar sections. (**C**) Fluorescent immunostaining of selected brain regions shown in A. Immunohistochemistry was performed using DAB staining in panels **A** and **B**, and fluorescent secondary antibodies (biotin-strepavidin amplification system) in panel **C** (magnification views). As indicated, ZNHIT3 expression is observed in the same regions as in panel A (Corpus callosum, internal and external capsules and optic tract). In the high magnified view, the signal is visible both in the cytoplasm and as punctate staining within the nucleus. Scale bars: 5 mm in the overview images, 100 μm in the magnified views and 10 μm in the high magnified view in panel C. IHC: immunohistochemistry, CC: corpus callosum, OT: optic tract, IC: internal capsule, EC: external capsule, 3V: third ventricle, 4V: fourth ventricle

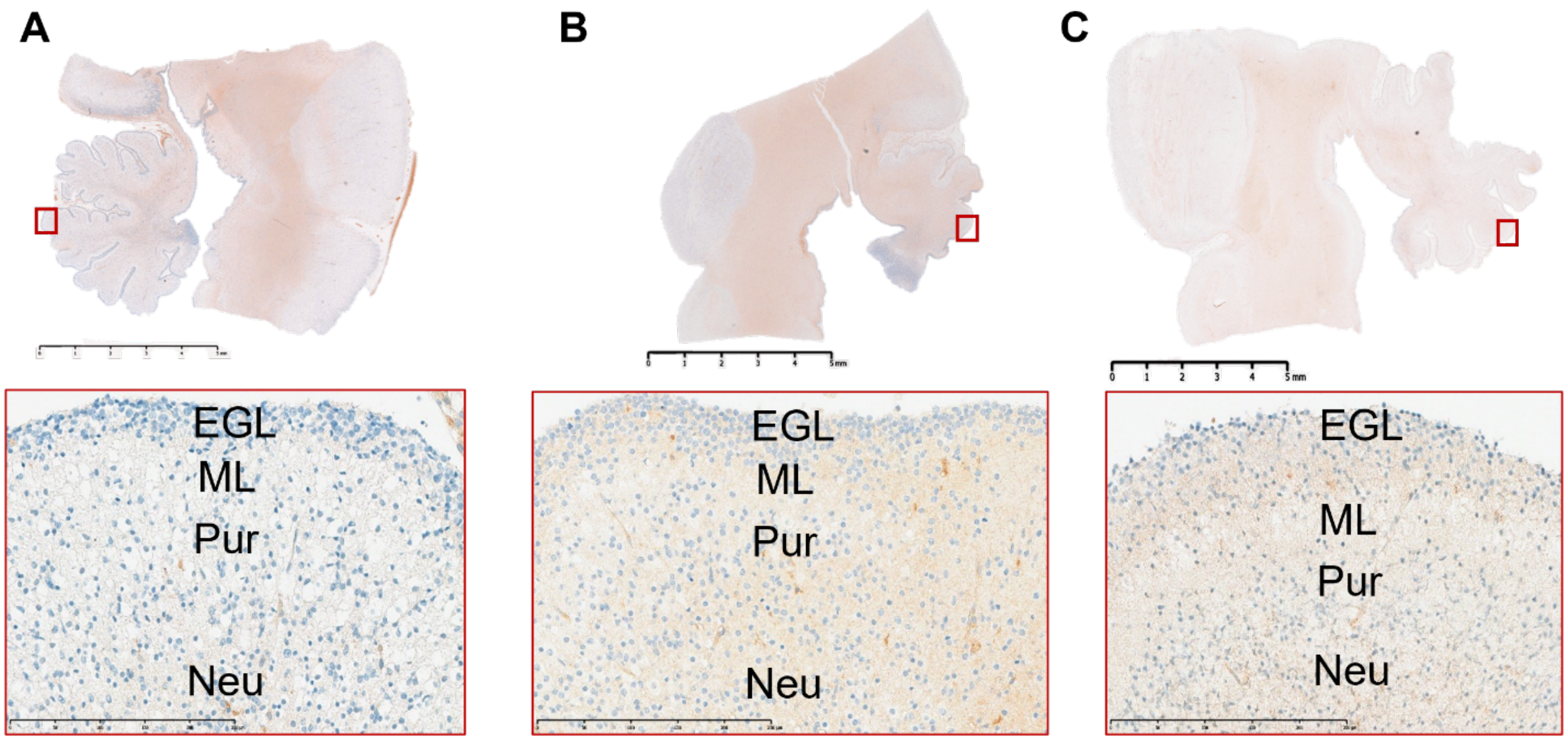

Figure S3. Immunohistochemical analysis of fetus samples. Immunohistochemical distribution of the ZNHIT3 protein in the cerebellum of the affected male fetus from the third pregnancy (**A**), control 1 at 15+4-GW (**B**), control 2 at 19+4 GW (**C**). External granular layer (EGL), thin molecular layer (ML), incomplete Purkinje layer (Pur), and migrated neuroblasts (Neu) are indicated. The internal granular layer was not detected.

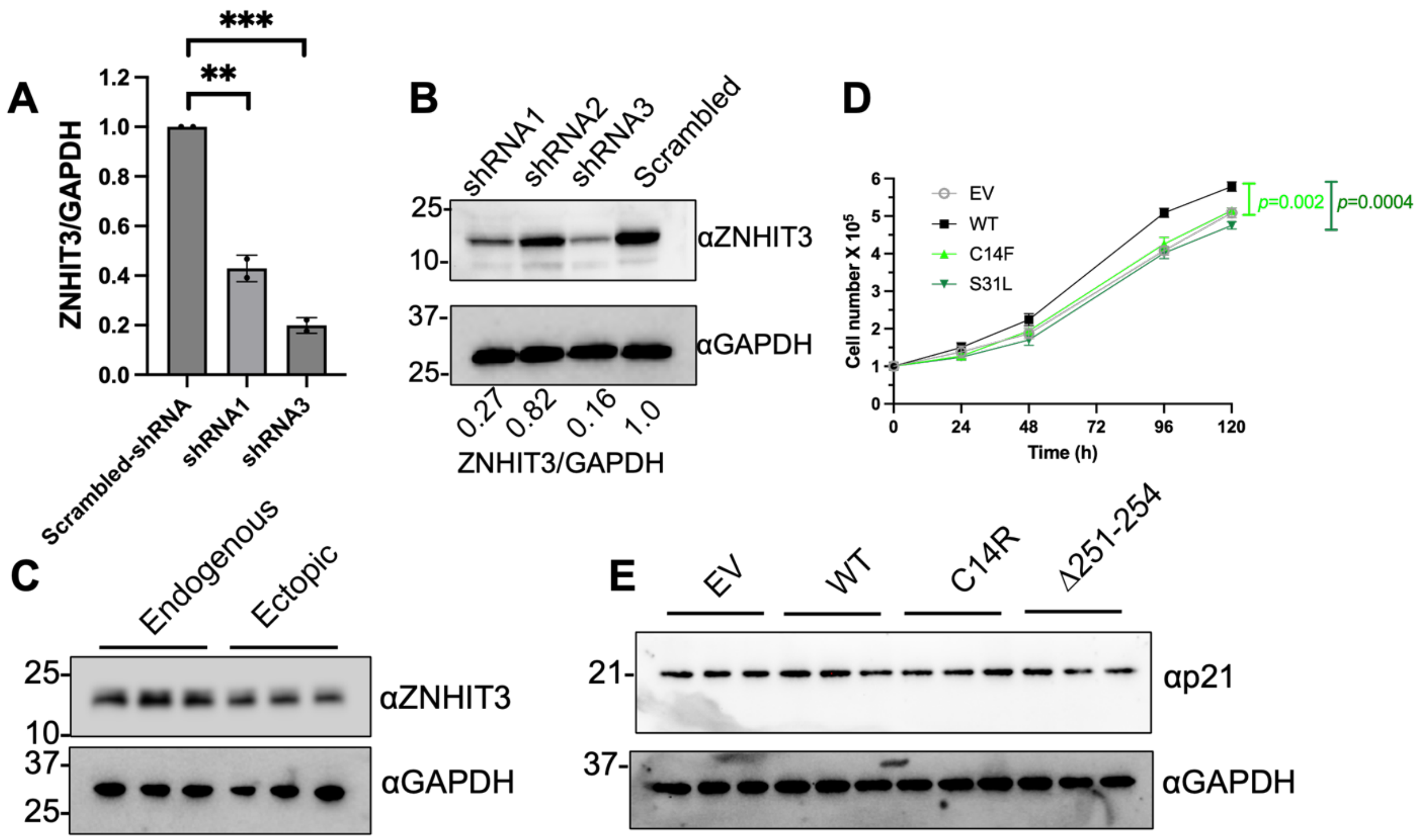

Figure S4. Establishing a ZNHIT3 knockdown cell line for studies of pathogenic ZNHIT3 variants. A) Relative expression of ZNHIT3 following shRNA knockdown in HEK293T cells was measured by RT-qPCR and normalized to endogenous GAPDH control transcript. Data are presented relative to ZNHIT3 expression in cells treated with scrambled shRNA. Bars represent mean ± SD from two independent biological replicates. **B)** Representative immunoblot analysis of ZNHIT3 knockdown HEK293T cells. Whole-cell lysates were analyzed using antibodies against ZNHIT3. GAPDH levels were measured as control. **C)** Representative immunoblot showing endogenous ZNHIT3 and ectopically expressed WT ZNHIT3 following stable cell line generation. Equal concentrations of protein from three independent biological replicates were analyzed by immunoblotting. GAPDH serves as a control. **D)** Cell growth assay of ZNHIT3-depleted cells expressing WT or previously reported variant ZNHIT3. Cell proliferation was monitored over the indicated time course and normalized to the initial measurement. Data represent mean ± SD from three independent biological replicates. p values were determined for the final timepoint by two-way ANOVA with multiple-comparison correction. **E)** Immunoblot analysis of p21 expression in ZNHIT3-depleted HEK293T cells complemented with WT or variant ZNHIT3. Representative immunoblots from three independent biological replicates are shown. GAPDH served as a loading control.

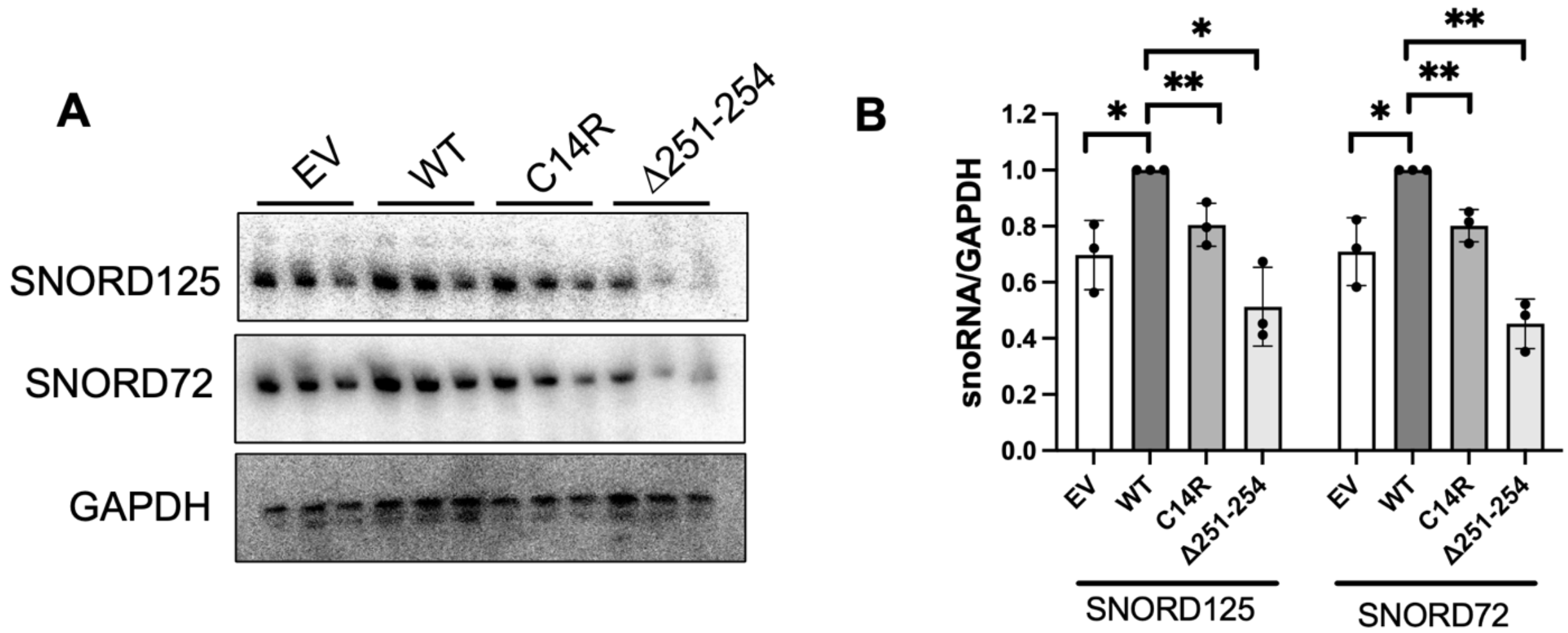

Figure S5. Validation of box C/D snoRNA abundance by northern blotting. A) Northern blot analysis of SNORD72 and SNORD125 in ZNHIT3-depleted HEK293T cells expressing WT or variant ZNHIT3. Equal amounts of total RNA from three independent biological replicates of each cell type were analyzed, and GAPDH mRNA served as the loading control. **B)** Quantification of SNORD72 and SNORD125 abundance normalized to GAPDH and shown relative to WT ZNHIT3-expressing cells. Bars represent the mean ± SD from three independent biological replicates.

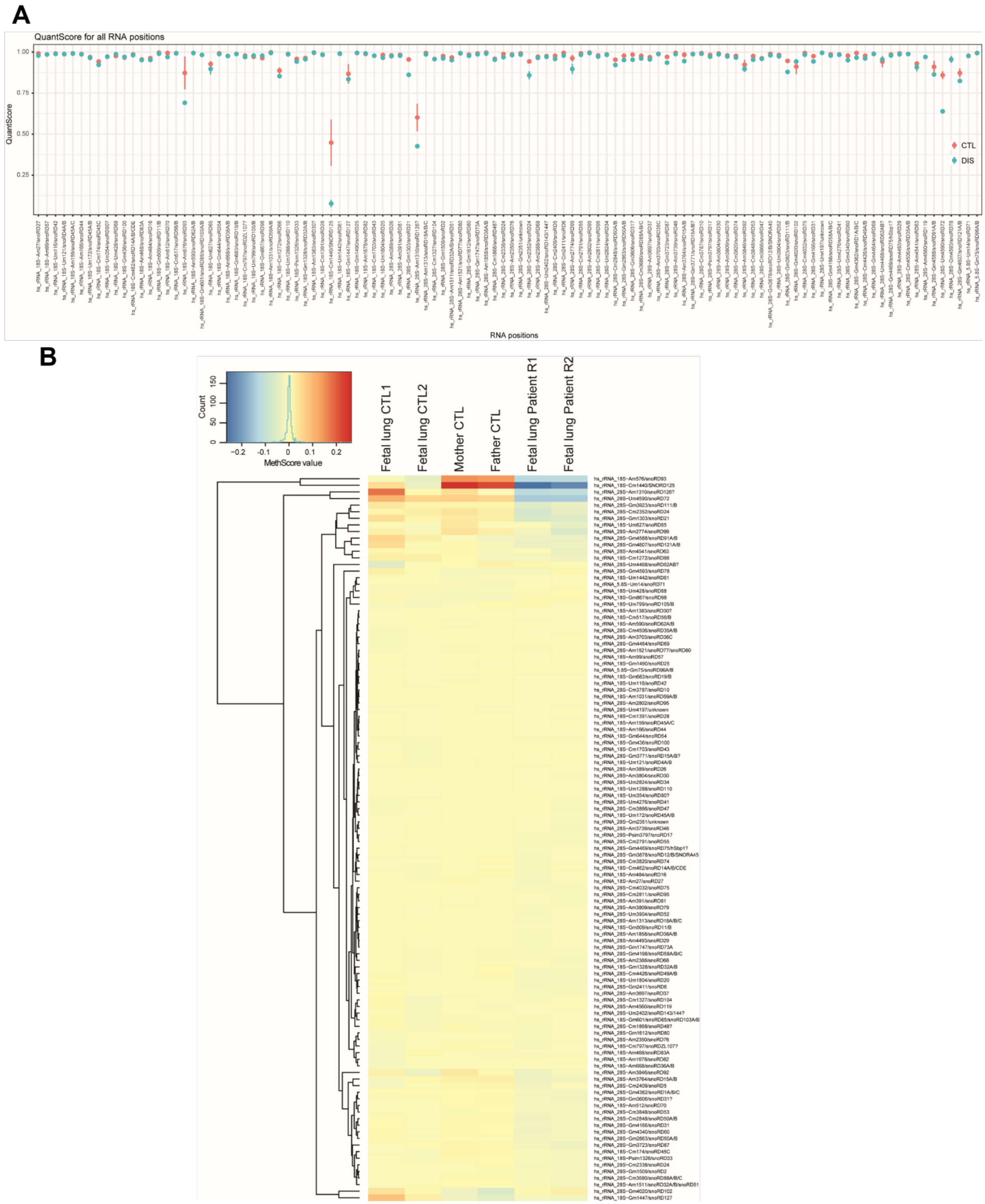

Figure S6. A) Human rRNA methylation level (indicated as MethScore) for all rRNA modification sites. Mean and S.D. are shown for two groups (CTL) and disease (DIS). The identity of the site and the corresponding C/D snoRNA is shown at the bottom. **B)** A full differential heat map showing MethScore variation for rRNA 2'-O-methylated positions in the fetus, parental samples, and fetal control samples is shown.

Table S1. List of plasmids used in this study.

Plasmid name	Backbone	Gene	Purpose
pRK5-EV	pRK5	*HA*	Overexpression
pRK5-HA-ZNHIT3 (Addgene #110953)	pRK5	*ZNHIT3*	Overexpression
pRK5-HA-ZNHIT3-C14R	pRK5	*ZNHIT3*	Overexpression
pRK5-HA-ZNHIT3-C14F	pRK5	*ZNHIT3*	Overexpression
pRK5-HA-ZNHIT3-S31L	pRK5	*ZNHIT3*	Overexpression
pRK5-HA-ZNHIT3-Δ251-254	pRK5	*ZNHIT3*	Overexpression
FUGW (Addgene #14883)	HR'CS-G	*EGFP*	Lentiviral expression
FUGW-ZNHIT3	HR'CS-G	*ZNHIT3*	Lentiviral expression
FUGW-ZNHIT3-C14R	HR'CS-G	*ZNHIT3*	Lentiviral expression
FUGW-ZNHIT3-Δ251-254	HR'CS-G	*ZNHIT3*	Lentiviral expression
pLVX_ZNHIT3-shRNA1	pLVX-shRNA1	shRNA	Lentiviral expression
pLVX_ZNHIT3-shRNA2	pLVX-shRNA1	shRNA	Lentiviral expression
pLVX_ZNHIT3-shRNA3	pLVX-shRNA1	shRNA	Lentiviral expression
pshRNA-NC	pLVX-shRNA1	shRNA	Lentiviral expression

Table S2. List of primers used in this study.

Primer	Sequence	Purpose
ZNHIT3_C14R	CGTCTGCGTGATCCGCTTGGAGAAGCC	Site directed mutagenesis
ZNHIT3_del251-254	GTGATGAGGAAGCAGAGTTTCTTTGC	Site directed mutagenesis
ZNHIT3_S31L	GCGTGCCCTACTGCTCCGTAGTCTGCTTCC	Site directed mutagenesis
ZNHIT3_C14F	CGTCTGCGTGATCTTCTTGGAGAAGCCCAA	Site directed mutagenesis
ZNHIT3_BamHI_For	CACCAAGGATCCATTCGCGCCACCATGGGCTA	Cloning
ZNHIT3-EcoRI-Rev	CTTACTTACGAATTCGGCCGCTTAAGACTCC	Cloning
SNORA30-For	GCACTTTCACAGTTCCTTCC	RT-qPCR
SNORA30-Rev	CAGGGCAAGAATACAATCAAGG	RT-qPCR
SNORA55-For	AGATGGTGCTACAGAATGAGC	RT-qPCR
SNORA55-Rev	AAATGTGGCAGAGCTGTAGAG	RT-qPCR
SNORA5A-For	CAGTACCTGTCCTATGCATGG	RT-qPCR
SNORA5A-Rev	ACCAAATTTATCCCTGAGCCT	RT-qPCR
SNORD91B-For	GTCTGAACCTGTCTGAAGCATCC	RT-qPCR
SNORD91B-Rev	AAGCCTCAGTATCACACAGAAGT	RT-qPCR
SNORD93-For	GCCAAGGATGAGAACTCTAATCTGA	RT-qPCR
SNORD93-Rev	GGCCTCAGGTAAATCCTTTAATCCA	RT-qPCR
SNORD114-13-For	GATCAATGATGACTACTGTT	RT-qPCR
SNORD114-13-Rev	GGACCTCAGAGTTTCAGACA	RT-qPCR
SNORD112-For	TGGACCAATGATGAGACAGTG	RT-qPCR
SNORD112-Rev	GTGCAGAACTGGATTAATCATG	RT-qPCR
SNORD125-For	CCCTCCTGATGATTCTTCTTCC	RT-qPCR
SNORD125-Rev	GTCAACTTCTTAGAGGCTCAGTT	RT-qPCR
SNORD3A-For	TAGCAGAGGTGTGCAAGGAGG	RT-qPCR
SNORD3A-Rev	GCAGTTGCAGCCAAGCAACG	RT-qPCR
SNORD14A-For	GATTGGTTGCCAGACATTCG	RT-qPCR
SNORD14A-Rev	CACTCAGACATCCAAGGAAG	RT-qPCR

SNORD18A-For	TAGTGATGAAATTCCACTTC	RT-qPCR
SNORD18A-Rev	CATCAGAACATCCGAGAAAA	RT-qPCR
SNORD21-For	GCTGAATGATGATATCCCAC	RT-qPCR
SNORD21-Rev	CTGCCATCAGTCCCGTCTTG	RT-qPCR
SNORD72-For	GCTTATCAGTGATGTTGTA	RT-qPCR
SNORD72-Rev	CGCTTATCTCAGTTAAATGC	RT-qPCR
GAPDH-For	CCACTCCTCCACCTTTGAC	RT-qPCR
GAPDH-Rev	ACCCTGTTGCTGTAGCCA	RT-qPCR
shRNA1_F’	GATCCGAGTCTTAAGATGGATTATTGTTCAAGAGA CAATAATCCATCTTAAGACTCTTTTTTG	shRNA construct
shRNA1_R’	AATTCAAAAAAGAGTCTTAAGATGGATTATTG TTCAAGAGA CAATAATCCATCTTAAGACTCG	shRNA construct
shRNA2_F’	GATCCACAGTTGATAGACATCATAAATTCAAGAGA TTTATGATGTCTATCAACTGTTTTTTTG	shRNA construct
shRNA2_R’	AATTCAAAAAA ACAGTTGATAGACATCATAAA TCTCTTGAA TTTATGATGTCTATCAACTGTG	shRNA construct
shRNA3_F’	GATCCCAGAGTTTCTTTGCAGAATTTTTCAAGAGA AAATTCTGCAAAGAAACTCTGTTTTTTG	shRNA construct
shRNA3_R’	AATTCAAAAAA CAGAGTTTCTTTGCAGAATTT TCTCTTGAAAAATTCTGCAAAGAAACTCTGG	shRNA construct
shRNA_Scrambled_F’	GATCCCCTAAGGTTAAGTCGCCCTCGCTCGAGCG AGGGCGACTTAACCTTAGGTTTTTG	shRNA construct
shRNA_Scrambled_R’	AATTCAAAAAACCTAAGGTTAAGTCGCCCTCGCTC GAGCGAGGGCGACTTAACCTTAGGGGATC	shRNA construct
5’-ETS	CGGAGGCCCAACCTCTCCGACGACAGGTCGCCA GAGGACAGCGTGTCAGC	Northern blot
ITS1	CCTCGCCCTCCGGGCTCCGGGCTCCGTTAATGAT C	Northern blot
ITS2	CTGCGAGGGAACCCCCAGCCGCGCA	Northern blot
28S	AACGATCAGAGTAGTGGTATTTCACC	Northern blot
18S	GCATGGCTTAATCTTTGAGACAAGCATAT	Northern blot
5.8S	CAATGTGTCCTGCAATTCAC	Northern blot
GAPDH	ACCCTGTTGCTGTAGCCA	Northern blot

Table S3. RNA modification scores.

	Fetal lung CTL1	Fetal lung CTL2	Mother CTL	Father CTL	Fetal lung Patient R1	Fetal lung Patient R2
hs_rRNA_18S-Am27/snoRD27	0.99	0.99	1.00	0.99	0.97	0.98
hs_rRNA_18S-Am99/snoRD57	0.99	0.98	0.99	0.99	0.99	0.99
hs_rRNA_18S-Um116/snoRD42	0.99	0.99	0.99	0.99	0.99	0.99
hs_rRNA_18S-Um121/snoRD4A/B	0.99	0.98	0.99	0.99	0.99	0.99
hs_rRNA_18S-Am159/snoRD45A/C	0.99	0.99	0.99	0.99	0.99	0.99
hs_rRNA_18S-Am166/snoRD44	0.99	0.98	0.99	0.99	0.99	0.99
hs_rRNA_18S-Um172/snoRD45A/B	0.96	0.96	0.97	0.98	0.97	0.96
hs_rRNA_18S-Cm174/snoRD45C	0.94	0.93	0.95	0.95	0.93	0.92
hs_rRNA_18S-Um354/snoRD90?	0.97	0.97	0.98	0.97	0.97	0.97
hs_rRNA_18S-Um428/snoRD68	0.98	0.98	0.98	0.97	0.99	0.99

hs_rRNA_18S-Gm436/snoRD100	0.97	0.96	0.97	0.97	0.97	0.97
hs_rRNA_18S-Cm462/snoRD14A/B/CDE	0.98	0.98	0.99	0.99	0.97	0.99
hs_rRNA_18S-Am468/snoRD83A	0.94	0.97	0.96	0.95	0.95	0.95
hs_rRNA_18S-Am484/snoRD16	0.96	0.95	0.97	0.97	0.95	0.96
hs_rRNA_18S-Gm509/snoRD11/B	1.00	1.00	1.00	1.00	0.99	0.99
hs_rRNA_18S-Am512/snoRD70	0.99	0.99	1.00	1.00	0.97	0.97
hs_rRNA_18S-Cm517/snoRD56/B	0.99	0.99	0.99	1.00	0.99	0.99
hs_rRNA_18S-Am576/snoRD93	0.80	0.77	0.96	0.95	0.69	0.70
hs_rRNA_18S-Am590/snoRD62A/B	0.99	1.00	0.99	1.00	0.99	0.99
hs_rRNA_18S-Gm601/snoRD85/snoRD103A/B	0.99	0.97	0.98	0.98	0.98	0.98
hs_rRNA_18S-Um627/snoRD65	0.92	0.91	0.95	0.92	0.92	0.87
hs_rRNA_18S-Gm644/snoRD54	0.99	0.99	0.99	0.99	0.99	0.99
hs_rRNA_18S-Am668/snoRD36A/B	0.96	0.98	0.98	0.98	0.98	0.98
hs_rRNA_18S-Gm683/snoRD19/B	0.99	0.99	0.99	0.99	0.99	0.99
hs_rRNA_18S-Cm797/snoRDZL107?	0.96	0.98	0.99	0.98	0.97	0.97
hs_rRNA_18S-Um799/snoRD105/B	0.97	0.96	0.97	0.99	0.99	0.97
hs_rRNA_18S-Gm867/snoRD98	0.98	0.96	0.96	0.96	0.98	0.98
hs_rRNA_18S-Am1031/snoRD59A/B	1.00	1.00	1.00	1.00	0.99	1.00
hs_rRNA_18S-Cm1272/snoRD66	0.90	0.90	0.89	0.86	0.86	0.85
hs_rRNA_18S-Um1288/snoRD110	0.99	0.99	0.99	0.99	0.99	0.99
hs_rRNA_18S-Psim1326/snoRD33	0.96	0.95	0.97	0.96	0.95	0.94
hs_rRNA_18S-Gm1328/snoRD32A/B	0.97	0.95	0.96	0.97	0.96	0.95
hs_rRNA_18S-Am1383/snoRD30?	1.00	1.00	1.00	1.00	1.00	1.00
hs_rRNA_18S-Cm1391/snoRD28	0.98	0.98	0.98	0.99	0.98	0.98
hs_rRNA_18S-Cm1440/SNORD125	0.37	0.29	0.59	0.54	0.06	0.09
hs_rRNA_18S-Um1442/snoRD61	0.99	0.99	0.99	0.99	0.99	0.99
hs_rRNA_18S-Gm1447/snoRD127	0.94	0.88	0.83	0.81	0.84	0.83
hs_rRNA_18S-Gm1490/snoRD25	0.99	0.99	0.99	0.99	0.99	1.00
hs_rRNA_18S-Am1678/snoRD82	0.98	0.99	0.99	0.99	0.99	0.99
hs_rRNA_18S-Cm1703/snoRD43	0.98	0.97	0.98	0.98	0.98	0.98
hs_rRNA_18S-Um1804/snoRD20	0.99	0.98	0.98	0.98	0.97	0.96
hs_rRNA_28S-Am389/snoRD26	0.97	0.98	0.98	0.98	0.98	0.97
hs_rRNA_28S-Am391/snoRD81	0.99	0.98	0.98	0.98	0.98	0.98
hs_rRNA_28S-Gm1303/snoRD21	0.97	0.94	0.95	0.95	0.86	0.86
hs_rRNA_28S-Am1310/snoRD126?	0.72	0.55	0.59	0.54	0.43	0.43
hs_rRNA_28S-Am1313/snoRD18A/B/C	0.99	0.99	0.99	0.99	0.98	0.99
hs_rRNA_28S-Cm1327/snoRD104	0.97	0.95	0.96	0.96	0.95	0.96
hs_rRNA_28S-Gm1509/snoRD2	0.97	0.97	0.98	0.98	0.96	0.96

hs_rRNA_28S-Am1511/snoRD32A/B/snoRD51	0.96	0.97	0.97	0.97	0.96	0.94
hs_rRNA_28S-Am1521/snoRD77/snoRD80	0.99	0.99	0.99	0.99	0.99	0.99
hs_rRNA_28S-Gm1612/snoRD80	0.98	0.98	0.99	0.99	0.97	0.98
hs_rRNA_28S-Gm1747/snoRD73A	0.99	0.99	0.99	0.99	0.98	0.99
hs_rRNA_28S-Am1858/snoRD38A/B	1.00	1.00	1.00	1.00	0.99	0.99
hs_rRNA_28S-Cm1868/snoRD48?	0.97	0.94	0.97	0.95	0.95	0.96
hs_rRNA_28S-Cm2338/snoRD24	0.99	0.99	0.99	0.99	0.97	0.97
hs_rRNA_28S-Am2350/snoRD76	0.98	0.99	0.99	0.99	0.98	0.99
hs_rRNA_28S-Gm2351/unknown	0.99	0.99	0.99	0.99	0.99	0.98
hs_rRNA_28S-Cm2352/snoRD24	0.93	0.94	0.95	0.94	0.84	0.88
hs_rRNA_28S-Am2388/snoRD68	0.97	0.97	0.98	0.97	0.97	0.96
hs_rRNA_28S-Um2402/snoRD143/144?	0.98	0.97	0.98	0.97	0.97	0.97
hs_rRNA_28S-Cm2409/snoRD5	0.96	0.98	0.99	0.99	0.95	0.97
hs_rRNA_28S-Gm2411/snoRD6	1.00	0.99	1.00	1.00	0.98	0.98
hs_rRNA_28S-Am2774/snoRD99	0.96	0.93	0.98	0.97	0.92	0.87
hs_rRNA_28S-Cm2791/snoRD55	0.99	0.99	1.00	0.99	0.98	0.98
hs_rRNA_28S-Am2802/snoRD95	1.00	1.00	1.00	1.00	0.99	0.99
hs_rRNA_28S-Cm2811/snoRD95	0.98	0.98	0.98	0.98	0.97	0.97
hs_rRNA_28S-Um2824/snoRD34	0.98	0.98	0.99	0.99	0.98	0.98
hs_rRNA_28S-Cm2848/snoRD50A/B	0.95	0.95	0.96	0.96	0.92	0.93
hs_rRNA_28S-Gm2863/snoRD50A/B	0.98	0.97	0.98	0.98	0.95	0.96
hs_rRNA_28S-Gm3606/snoRD31?	0.98	0.98	0.99	0.99	0.95	0.95
hs_rRNA_28S-Cm3680/snoRD88A/B/C	0.97	0.97	0.98	0.98	0.97	0.96
hs_rRNA_28S-Am3697/snoRD37	0.97	0.96	0.97	0.97	0.96	0.95
hs_rRNA_28S-Am3703/snoRD36C	0.98	0.99	0.99	0.99	0.99	0.99
hs_rRNA_28S-Gm3723/snoRD87	0.96	0.97	0.98	0.98	0.94	0.93
hs_rRNA_28S-Am3739/snoRD46	0.99	1.00	0.99	1.00	0.99	0.98
hs_rRNA_28S-Am3764/snoRD15A/B	0.96	0.99	0.99	1.00	0.94	0.95
hs_rRNA_28S-Gm3771/snoRD15A/B?	0.99	0.98	0.99	0.99	0.98	0.99
hs_rRNA_28S-Cm3787/snoRD10	0.99	0.99	0.99	0.99	0.99	0.99
hs_rRNA_28S-Psim3797/snoRD17	0.98	0.99	0.99	0.98	0.97	0.97
hs_rRNA_28S-Am3804/snoRD30	0.99	0.99	0.99	0.99	0.99	0.98
hs_rRNA_28S-Am3809/snoRD79	0.98	0.97	0.98	0.98	0.97	0.97
hs_rRNA_28S-Cm3820/snoRD74	0.97	0.97	0.98	0.98	0.96	0.97
hs_rRNA_28S-Am3846/snoRD92	0.94	0.88	0.95	0.92	0.88	0.91
hs_rRNA_28S-Cm3848/snoRD53	0.97	0.97	0.98	0.98	0.95	0.95
hs_rRNA_28S-Cm3866/snoRD47	0.96	0.96	0.96	0.97	0.97	0.95
hs_rRNA_28S-Gm3878/snoRD12/B/SNORA45	0.99	0.99	0.99	0.99	0.98	0.99

hs_rRNA_28S-Um3904/snoRD52	0.99	0.98	0.98	0.98	0.97	0.98
hs_rRNA_28S-Gm3923/snoRD111/B	0.96	0.94	0.94	0.94	0.87	0.89
hs_rRNA_28S-Gm4020/snoRD102	0.96	0.93	0.89	0.86	0.94	0.95
hs_rRNA_28S-Cm4032/snoRD75	1.00	1.00	0.99	0.99	0.98	0.98
hs_rRNA_28S-Gm4166/snoRD31	0.98	0.97	0.98	0.98	0.94	0.95
hs_rRNA_28S-Um4197/unknown	1.00	1.00	1.00	0.99	0.99	1.00
hs_rRNA_28S-Gm4198/snoRD58A/B/C	0.99	0.98	0.99	0.98	0.98	0.98
hs_rRNA_28S-Um4276/snoRD41	0.98	0.99	0.99	0.99	0.99	0.98
hs_rRNA_28S-Gm4340/snoRD60	0.98	0.97	0.98	0.98	0.94	0.96
hs_rRNA_28S-Gm4362/snoRD1A/B/C	0.99	0.99	0.99	1.00	0.97	0.96
hs_rRNA_28S-Cm4426/snoRD49A/B	0.98	0.97	0.98	0.98	0.96	0.96
hs_rRNA_28S-Gm4464/snoRD69	0.98	0.99	0.99	0.99	0.99	0.99
hs_rRNA_28S-Um4468/snoRD62AB?	0.89	0.95	0.96	0.97	0.96	0.95
hs_rRNA_28S-Gm4469/snoRD75/hSbp1?	0.99	0.98	0.98	0.99	0.98	0.98
hs_rRNA_28S-Am4493/snoRD29	0.99	0.99	0.99	0.99	0.98	0.99
hs_rRNA_28S-Cm4506/snoRD35A/B	0.98	0.99	0.99	0.99	0.99	0.99
hs_rRNA_28S-Am4541/snoRD63	0.94	0.93	0.94	0.91	0.93	0.89
hs_rRNA_28S-Am4560/snoRD119	0.99	0.96	0.97	0.97	0.97	0.97
hs_rRNA_28S-Gm4588/snoRD91A/B	0.96	0.92	0.89	0.87	0.87	0.86
hs_rRNA_28S-Um4590/snoRD72	0.90	0.85	0.85	0.84	0.64	0.64
hs_rRNA_28S-Gm4593/snoRD78	0.98	0.96	0.94	0.94	0.94	0.97
hs_rRNA_28S-Gm4607/snoRD121A/B	0.91	0.86	0.87	0.85	0.82	0.83
hs_rRNA_5.8S-Um14/snoRD71	0.98	0.98	0.97	0.97	0.98	0.98
hs_rRNA_5.8S-Gm75/snoRD96A/B	0.99	0.99	0.99	0.99	0.99	0.99
